# Dose–response prediction for in-vitro drug combination datasets: a probabilistic approach

**DOI:** 10.1186/s12859-023-05256-6

**Published:** 2023-04-21

**Authors:** Leiv Rønneberg, Paul D. W. Kirk, Manuela Zucknick

**Affiliations:** 1 Oslo Centre for Biostatistics and Epidemiology, University of Oslo, Oslo, Norway; 2 MRC Biostatistics Unit, University of Cambridge, Cambridge, UK; 3 Cambridge Institute of Therapeutic Immunology and Infectious Disease, University of Cambridge, Cambridge, UK; 4 Ovarian Cancer Programme, Cancer Research UK Cambridge Centre, Cambridge, UK

**Keywords:** Gaussian process regression, Bayesian inference, Cell viability assay, Drug synergy

## Abstract

In this paper we propose PIICM, a probabilistic framework for dose–response prediction in high-throughput drug combination datasets. PIICM utilizes a permutation invariant version of the intrinsic co-regionalization model for multi-output Gaussian process regression, to predict dose–response surfaces in untested drug combination experiments. Coupled with an observation model that incorporates experimental uncertainty, PIICM is able to learn from noisily observed cell-viability measurements in settings where the underlying dose–response experiments are of varying quality, utilize different experimental designs, and the resulting training dataset is sparsely observed. We show that the model can accurately predict dose–response in held out experiments, and the resulting function captures relevant features indicating synergistic interaction between drugs.

**Supplementary Information:**

The online version contains supplementary material available at 10.1186/s12859-023-05256-6.

## Introduction

Utilizing combinations of drugs to treat cancer is commonplace in modern treatment regimes offered to patients [1, 2]. Drug combinations have multiple benefits over single drug regimens, offering increased survival and treatment efficacy for patients, and at the same time allowing a lower total drug burden, in turn lessening side-effects [3, 4]. Combinations can also have the effect of minimizing the chances of the tumour acquiring drug resistance [5]. Finally, by utilizing novel targeted drugs and monoclonal antibodies in combinations, treatments can be personalised to the individual patient’s disease, exploiting weaknesses in the cancer cells while leaving normally functioning cells unharmed, through e.g. synthetic lethality [6].

When looking for effective drug combinations, researchers have often focused on the notion of drug synergy. That is, a drug combination whose joint effect is greater than what would be expected by using the two drugs individually. Synergistic combinations offer significant boosts in efficacy, at lower doses than would be needed had the drugs been administered on their own. The opposite effect, where two drugs perform worse together than would be expected is called antagonism, and finally if neither synergy nor antagonism is present in a combination, we say that the drugs are non-interacting [7].

The nature of a drug combination, as synergistic, antagonistic or non-interacting, is usually studied by in-vitro dose–response experiments on cancer cell lines, through e.g. a cell viability assay [8]. In these experiments, cancer cells are treated with multiple concentrations of the component drugs individually and in combination, and viability is measured by comparing a marker associated with viability (e.g. ATP levels) to negative and positive controls. Further processing takes place to determine if a drug combination is either synergistic, antagonistic or non-interacting. With modern high-throughput technology, thousands of drug combinations can quickly be evaluated in parallel on multiple cell lines in-vitro, aiding in synergistic drug combination discovery.

In recent years, several such datasets have been made available to researchers [9–11], with the aim of understanding mechanisms of drug synergy, as well as being used as input in machine learning algorithms predicting drug synergy or efficacy of drug combinations in new cell lines or patients. Most often, the input in these algorithms is a scalar measure of drug interaction, often called a synergy score, derived from the dose–response surface.The goal is to predict the synergy score in unseen experiments, either on untested cell lines or for completely novel, previously unobserved drug combinations (see e.g. [12] for a review of these approaches to synergy prediction, including various deep learning approaches such as [13, 14]).

There are a few major drawbacks with this current practice of drug synergy prediction. Firstly, there is no commonly agreed upon and generally applicable definition of drug synergy. Quantifying drug synergy relies on first settling on a model for non-interaction, essentially defining what non-interaction looks like, i.e. what would be expected if the drugs do not interact. Several such definitions have been made, each encoding its own pharmacological assumptions, the two most popular ones being the Bliss independence model [15] and Loewe additivity [16] (several modern approaches exist as well, see e.g. [17–19]). Exactly which model is deemed most correct depends on the drugs in question, and their mechanism of action. Secondly, once the preferred model of non-interaction has been chosen, it is common to quantify interaction point-wise as the difference between the observed and expected cell viability at each concentration combination. The problem with this approach is that it ignores the underlying measurement error and treats any deviance from the non-interaction model as evidence of drug interaction. Because of the heterogeneity of cell growth, as well as technical error sources, the noise component of the individual viability measurement can be considerable, and will vary across the dose–response surface [20, 21]. The underlying noise and measurement error has been largely ignored in the literature, where synergy scores are usually taken as precisely measured quantities, or measured with an identical noise across experiments [14, 22]. Finally, the synergy score used for training models is typically produced by taking an average across all observed concentrations, further obscuring the complex landscape of the dose–response surface which can contain several local minima and maxima.

Because of the problems associated with the synergy scores, a recent trend has been to directly predict the dose–response surface, and derive the synergy score as a post-processing step if desired.

Notably, the comboFM model [23] makes use of a higher-order factorization machine to predict dose–response for drug combinations. The comboFM model is able to handle the large and sparse reality of drug combination datasets, and can easily incorporate various information sources regarding the cell lines or the drugs involved, examples of which could be molecular descriptors of the drugs and various *omics* datasets for the cancer cell lines. Recently, comboFM was improved upon by a new algorithm, comboLTR [24], which uses a latent tensor reconstruction method as its main workhorse, that allows a more general form of its linear model, and is more computationally efficient than comboFM. There is also the IDACombo framework of [25], that employs the principle of Independent Drug Action to predict dose–response of combinations. The principle stipulates that the expected combined effect of multiple drugs administered together equals the effect of the single most effective drug in the combination administered on its own. Thus, the framework only utilizes monotherapy measurements of response for its predictions and does provides the key insight that most drug combination experiments are fit remarkably well by relatively simple models based solely on monotherapy measurements. The IDACombo framework is however quite limited in its simplicity, and is not able to incorporate e.g. other information sources regarding the cancer cell lines or the drugs. Additionally, the framework only provides an *averaged prediction* across multiple cell lines and does not provide a unique *per cell-line* prediction of response, further hindering its performance and wider applicability.

While these models directly predict dose–response and hence avoid the problems associated with the synergy scores, they still treat the measured viability percentages as precisely measured quantities, ignoring the heteroskedastic measurement error and inherent uncertainty of the underlying experiments.

Recently, Tansey et al. [21] proposed an end-to-end approach to dose–response modelling that naturally incorporates measurement error. Starting from raw fluorescent counts directly from the plate-reader, a large Bayesian hierarchical model was set up to describe the entire data-generating process. In this fashion, the measurement uncertainty coming from both biological and technical sources is handled properly, allowing for cleaner estimates of the dose–response function. The individual experiments’ dose–response functions are further linked to drug and cell line auxiliary data through a neural network. In this way the authors are able to uncover both well-known and novel biomarkers of drug response, and improve the state of the art in terms of prediction performance on held out experiments. However, the model is tailored for single drug responses, and impose restrictions, such as monotonicity, that could make it unfeasible for drug combination data.

Predicting the full dose–response surface for drug combinations brings an additional challenge. Since the drugs involved in each combination can be ordered in any manner, the predicted dose–response should be exactly the same no matter which way the drugs in a pair are ordered. That is, if we denote by $f(c,(A,B),(x_A,x_B))$ the predicted dose–response of the drug combination (*A*, *B*) at concentrations $(x_A,x_B)$ in cell line *c*, the model should predict the exact same output if the ordering was reversed:

1 $$\begin{aligned} f(c,(A,B),(x_A,x_B)) = f(c,(B,A),(x_B,x_A)). \end{aligned}$$

In a supervised learning setting, there are two main ways to deal with this type of symmetry, or *invariance*. The easiest path is to augment the initial training dataset by adding examples that have undergone transformation known to leave the output unchanged. For drug combination datasets, this would amount to doubling the training data, including both orderings of the drug pairs for each experiment. By training a suitably flexible model, data augmentation encourages invariant parametrizations of the model, but does not enforce it. This is the the approach taken by both the comboFM and the comboLTR model. However, this is unsatisfactory from a Bayesian viewpoint where all the relevant assumptions should be built into the prior. Duplicating the data could also lead to the posterior variance being too narrow, and the model being too confident in its predictions. The other way, which is more preferable from a Bayesian perspective, is to directly encode the invariance into the model construction. Building invariant models has been an empirical success story particularly within deep learning, starting from the early convolutional neural nets made to classify handwritten digits [26]. In these models, the outcome is made invariant to translations of the input image, often with considerable boosts in prediction performance compared to non-invariant models [27, 28]. There is also an emerging theoretical understanding of *why* models that directly encode invariance can often perform better than augmenting the training data, particularly in settings where the data is either of low-quality or scarse [29, 30].

In this paper we introduce PIICM, a probabilistic prediction model based on a Permutation Invariant Intrinsic Coregionalization model for multi-output Gaussian Process (GP) regression, that aims to alleviate some of the issues associated with the current methodological landscape of drug combination prediction. By developing a permutation-invariant, multi-output GP prior, where each drug combination experiment is considered an output, the relevant invariances for dose–response prediction are built directly into the model, thus avoiding the issues associated with data augmentation. Furthermore, a two-stage observation model is utilized that incorporates the measurement error within and between individual experiments, accounting for the potential differences in experimental quality. The GP model is implemented in a computationally efficient manner built on top of the GPyTorch [31] library, which allow fast and exact inference with GPU acceleration.

## Methods

The main model proposed in this paper builds on the bayesynergy [20] model for dose–response estimation of individual two-drug combination experiments. Our main idea here is to extend this single-experiment dose–response model to a model that accounts for correlations between dose–response functions from different experiments. The way this is achieved is by linking together the latent GPs underlying each dose–response function by explicitly modelling the cross-function covariance in a multi-output framework. The multi-output framework can be used to predict the values of the latent GP in unobserved experiments, which are then used to reconstruct the full dose–response function of that experiment.

**Fig. 1:**
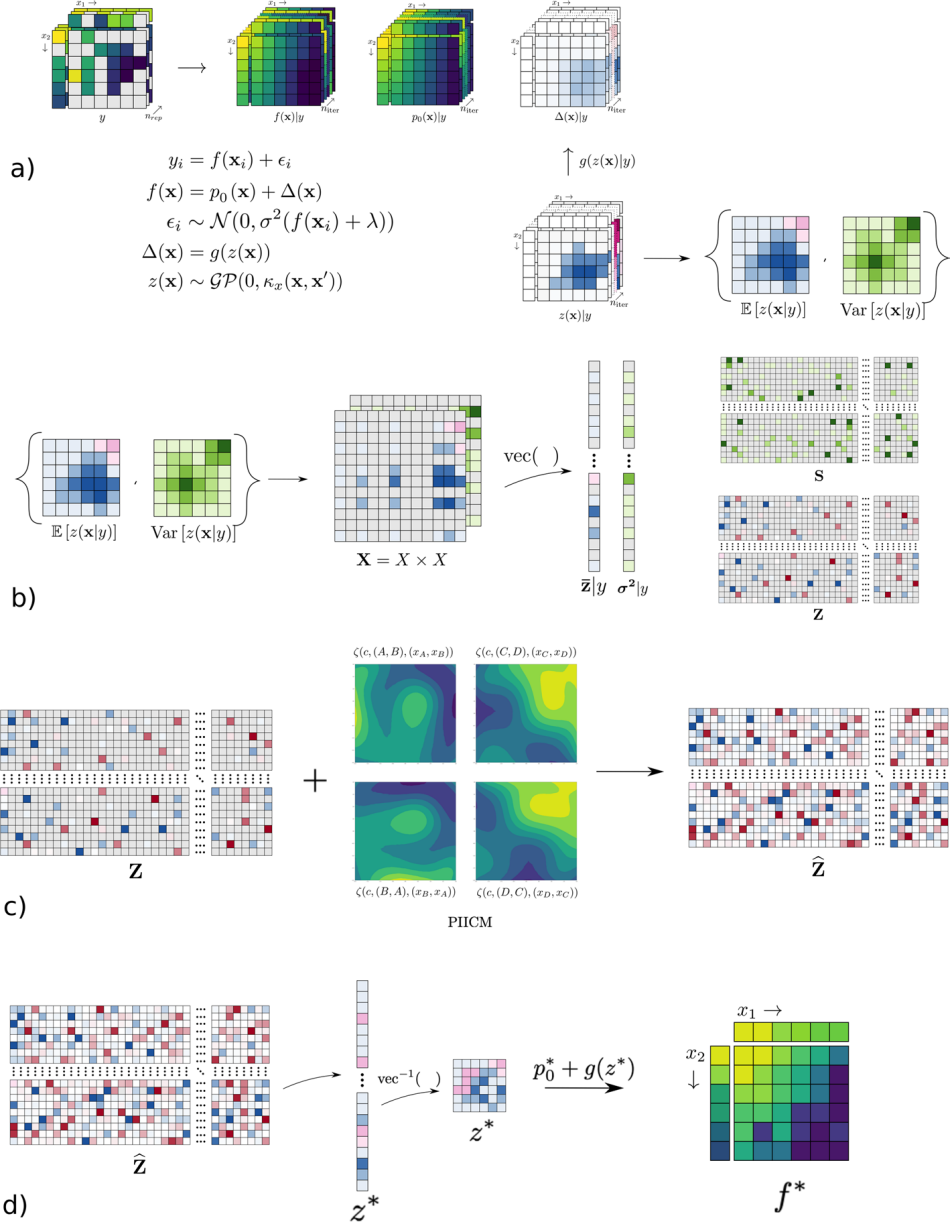
The figure shows the overall workflow of the method. In (**a**) the bayesynergy R package [20] is used as a pre-processing step to estimate the latent GP underlying each experiment, which is summarized by its posterior mean and variance. In (**b**), the posterior mean and variance of each experiment is put into a common grid, and collected in matrices ${\textbf{Z}}$ and ${\textbf{S}}$. In (**c**), the missing entries of ${\textbf{Z}}$ is predicted using the PIICM model. And finally, in (**d**) the predicted latent GP of an unseen experiment is used to reconstruct the dose–response function

In the following we first introduce in some detail the bayesynergy dose–response model, highlighting how the latent GP enters in the modelling framework and the extension to the multi-output setting through the intrinsic coregionalization model (ICM) [32]. We then detail the construction of a permutation invariant ICM kernel, which encodes the required invariances for drug combination experiments. Next, the observation model is introduced where we show how individual level observation noise can be introduced through the likelihood to account for differences in experimental uncertainty. The procedure for inference and parameter learning is introduced in the “Inference and parameter learning” ﻿section, where we show how the model handles incomplete datasets through a masking technique. Finally, we show how the dose–response function in unseen experiments can be reconstructed using the predicted values of the corresponding latent GP. The general workflow of the model is illustrated in Fig. 1.

### Bayesian dose–response model for single experiments

The main object of interest in drug sensitivity screens is the dose response function, which maps a drug concentration *x* to cell viability, i.e. taking values in the interval [0, 1] where zero indicates all the cells are dead, and one that all cells are still viable. In monotherapy screens, where only a single drug is tested at a time, it is common to employ a log-logistic model for the dose–response function:

2 $$\begin{aligned} h(x) = l + \frac{u-l}{1+10^{s(x-m)}}, \end{aligned}$$

where *x* is the drug concentration on the $\log _{10}$ scale. The parameters (*u*, *l*) denote the upper and lower asymptotes representing the minimum and maximum effect of the drugs on the cell lines, respectively. The parameter *s* controls the slope of the function, how quickly the drug reaches maximum effect with increasing concentration. And finally the *m* parameter is the *half maximal effective concentration*, or $\text{EC}_{50}$, acting as a location parameter giving the concentration at which the drug reaches half of its maximum effect. Because we are usually working with normalized viability measurements, and assume no drugs are beneficial to cell growth, the upper asymptotes are fixed at one throughout the paper.

In drug combination screens, it is common to represent the dose–response function by two parts: a non-interaction term $p_0$ capturing a non-interaction assumption, and an interaction term $\Delta$ capturing any additional effect, synergistic or antagonistic:

3 $$\begin{aligned} f({\textbf{x}}) = p_0({\textbf{x}})+\Delta ({\textbf{x}}), \end{aligned}$$

where ${\textbf{x}}=(x_1,x_2)$ denotes the concentrations of the two drugs. For the non-interaction term, we utilize the Bliss independence assumption [15] which states that the joint effect of two drugs is simply the product of the two drugs’ individual effects – each modelled parametrically through Eq. (2):

4 $$\begin{aligned} p_0({\textbf{x}})=h_1(x_1)h_2(x_2), \end{aligned}$$

where each monotherapy function is estimated with its own set of parameters $(l_i,s_i,m_i)$ for $i=1,2$. There are several possible choices for the non-interaction term, e.g. Loewe additivity [16] or the *zero interaction potency* (ZIP) model [33]. While we utilize the Bliss model due to its simplicity and ease of calculation, this choice only functions as a jumping-off point for the larger goal of modelling the full response surface.

The interaction term is modelled using a latent Gaussian Process (GP) formulation. A GP is a stochastic process, any finite realization of which takes the form of a multivariate normal distribution. That is if $z\sim \mathcal{G}\mathcal{P}(\mu ({\textbf{x}}),\kappa ({\textbf{x}},{\textbf{x}}'))$ then

5 $$\begin{aligned} \left[ z({\textbf{x}}_1),\ldots ,z({\textbf{x}}_n)\right] ^T \sim \mathcal{MVN}(\varvec{\mu },K), \end{aligned}$$

where $\varvec{\mu }=\left[ \mu ({\textbf{x}}_1),\ldots ,\mu ({\textbf{x}}_n)\right] ^T$ is the mean vector and the entries of the covariance matrix *K* are $K_{ij}=\kappa ({\textbf{x}}_i,{\textbf{x}}_j)$. The function $\kappa (\cdot ,\cdot )$ is called the kernel function, and is of particular importance as it controls many features of the resulting function, such as smoothness. The GP is fully specified by its mean and covariance functions.

In order to ensure the correct bounds for the resulting dose–response function, i.e. that $f({\textbf{x}})\in [0,1]$, the latent GP is pushed through a transformation function to give the interaction surface $\Delta$. In detail

6 $$\begin{aligned} \begin{aligned} \Delta ({\textbf{x}}) = g(z({\textbf{x}})) \\ z \sim \mathcal{G}\mathcal{P}(0,\kappa ({\textbf{x}},{\textbf{x}}')) \\ g({\textbf{x}}) = \frac{-p_0({\textbf{x}})}{1+\exp \left[ b_1z({\textbf{x}})+\log \left( \frac{p_0({\textbf{x}})}{1-p_0({\textbf{x}})}\right) \right] } \\+ \frac{1-p_0({\textbf{x}})}{1+\exp \left[ -b_2z({\textbf{x}})-\log \left( \frac{p_0({\textbf{x}})}{1-p_0({\textbf{x}})}\right) \right] }, \end{aligned} \end{aligned}$$

where $(b_1,b_2)$ are parameters needed to keep the model identifiable, and $\kappa$ is the kernel function for the underlying GP. In addition to ensuring the correct bounds for the dose–response function *f*, the transformation function $g(\cdot )$ acts to encode an expectation on the interaction term $\Delta$. If the model is uncertain, or is extrapolating far outside the range of the data, the underlying GP will revert to its prior mean at zero, which when plugging in $g(0)=0$, entails that the dose–response function returns to $f({\textbf{x}})=p_0({\textbf{x}})$ in the case of lack of data. Generally, since large interaction effects are rare [34–36], the non-interaction assumption is often a reasonable approximation to the full dose–response function. A full model description including the prior distributions for all parameters is given in the Additional file 1. See also the original paper implementing this model [20] for more details.

### Joint modelling of latent GPs

The model from the previous section can be extended to take into account the correlation between individual dose–response functions. Specifically, the individual latent GPs underlying each experiment’s dose–response function can be estimated jointly using a multi-output GP framework. In a multi-output GP setting, the GP is extended from single valued to vector-valued outputs. In our context, every output corresponds to a different dose–response experiment, each consisting of a cell line and two drugs in a combination. Many approaches exist for multi-output GPs, see e.g. [32] for a review, and we opt for the simple intrinsic coregionalization model (ICM), that we extend to a permutation invariant version.

In the ICM, the covariance between two function evaluations $z_i({\textbf{x}})$ and $z_j({\textbf{x}}')$ is written as a product between an output kernel and an input kernel:

$$\begin{aligned} \text{Cov}\left[ z_i({\textbf{x}}),z_j({\textbf{x}}')\right]&= \text{Cov}\left[ z_i,z_j\right] \text{Cov}\left[ {\textbf{x}},{\textbf{x}}'\right] \\&= \kappa _{\text{z}}(z_i,z_j)\kappa _{\text{x}}({\textbf{x}},{\textbf{x}}'), \end{aligned}$$

where the input kernel $\kappa _{\text{x}}$ controls the smoothness of the functions across input space as in the regular single-output setting of “Bayesian dose–response model for single experiments” section, and the output kernel $\kappa _{\text{z}}$ explicitly models the covariance between the functions, or outputs. If it is the case that every input is evaluated on every output, then this induces a Kronecker structure on the covariance matrix of the joint multivariate normal. That is, if we have a function with *m* outputs evaluated at *n* distinct inputs, and assume a mean zero GP, the joint vector of responses has a zero-mean multivariate normal distribution;

7 $$(z_1({\textbf{x}}_1),\ldots ,z_1({\textbf{x}}_n),\ldots , z_m({\textbf{x}}_1)\ldots ,z_m({\textbf{x}}_n))^T \sim \mathcal{MVN}(0,K_{\text{output}} \otimes K_{\text{x}}),$$

where $K_{\text{output}}$ is an $m\times m$ matrix corresponding to the output covariance, i.e. with entries $K_{\text{output},ij}=\kappa _{\text{z}}(z_i,z_j)$ and $K_{\text{x}}$ an $n \times n$ matrix of input covariance constructed using the corresponding input kernel. Note that we can write the expression above more succinctly as

8 $$\begin{aligned} \text{vec}({\textbf{Z}}) \sim \mathcal{MVN}(0,K_{\text{output}} \otimes K_{\text{x}}), \end{aligned}$$

where ${\textbf{Z}}$ is the $n \times m$ matrix constructed such that the *i*-th column corresponds to the vector $(z_i({\textbf{x}}_1),\ldots ,z_i({\textbf{x}}_n))^T$, and $\text{vec}()$ denotes the operator that creates a column vector by stacking each column of the matrix on top of each other.

Because of the large number of experiments typically performed in high-throughput drug combination screens, we impose further structure on the output covariance in order to keep the model computationally feasible. Let $z_{cAB}$ denote the latent GP underlying the experiment consisting of the drug combination (*A*, *B*) and cell line *c*, and let $z_{c'A'B'}$ similarly denote the latent GP corresponding to another experiment of the drug combination $(A',B')$ on cell line $c'$. We assume that the covariance between two outputs (experiments) can be decomposed into a covariance across cell lines and a covariance across drug combinations:

9 $$\begin{aligned} \kappa _z(z_{cAB},z_{c'A'B'})=\kappa _c(c,c')\kappa _d((A,B),(A',B')). \end{aligned}$$

Similarly to before, if we assume that each drug combination is screened on every cell line, the output kernel matrix can also be written as a Kronecker product and:

10 $$\begin{aligned} \text{vec}({\textbf{Z}}) \sim \mathcal{MVN}(0,K_c\otimes K_d \otimes K_x), \end{aligned}$$

where $K_{c}$ is an $N_c \times N_c$ covariance matrix over cell lines, and $K_{d}$ the $N_d \times N_d$ covariance matrix over drug combinations. The columns of the $n\times (N_cN_d)$ matrix ${\textbf{Z}}$ must be ordered such that the first $N_d$ columns correspond to the first cell line, the next $N_d$ to the second, and so on.

While this structure is simplistic, similarly decomposed covariance structures have been utilized successfully for single-drug predictions previously [37].

#### A permutation invariant ICM

**Fig. 2:**
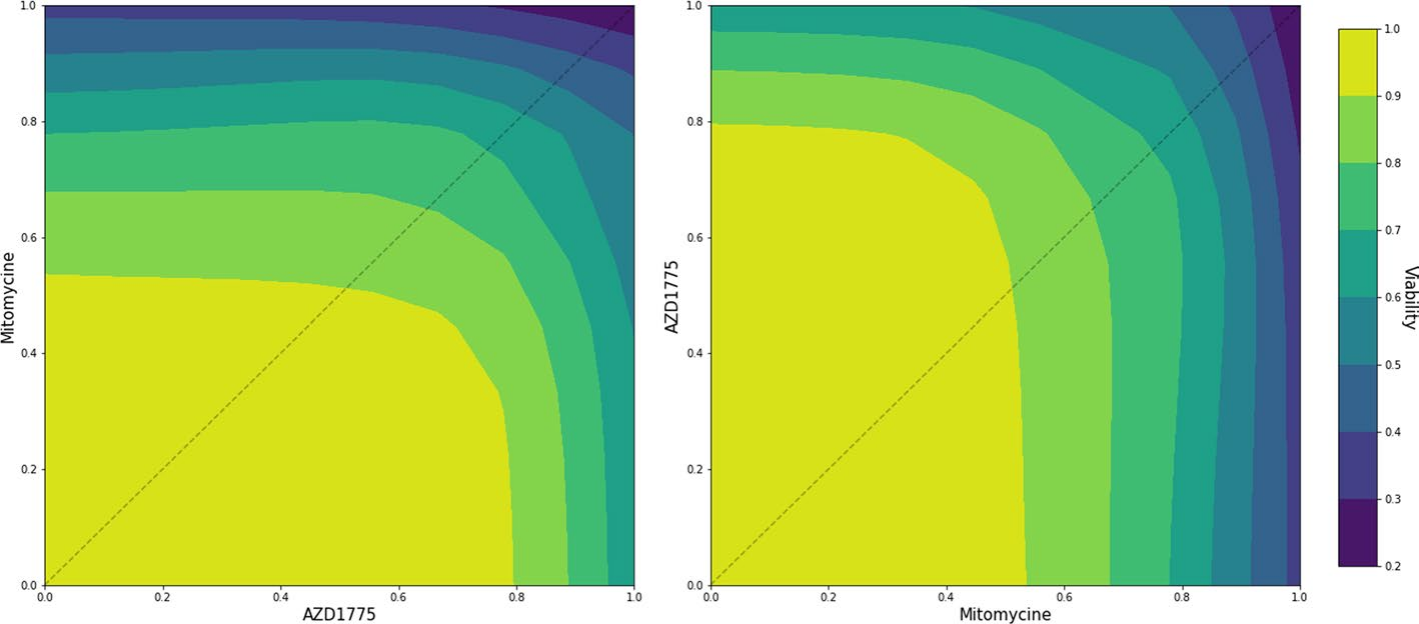
The figure shows the estimated dose–response surfaces for the two drugs AZD1775 and Mitomycine on the HT144 malignant melanoma cell line, taken from the [10] dataset. Depending on which drug is plotted on the *x*-axis, the dose–response surface flips around the 45$^\circ$ line

As previously discussed, the dose–response functions for drug combinations have some natural symmetries and invariances to consider. Letting $f(c,A,B,{\textbf{x}})$ denote the drug response for the drug combination (*A*, *B*), at concentration ${\textbf{x}}=(x_{1},x_{2})$, on cell line *c*. The dose–response is invariant to a certain permutation of its inputs, namely:

11 $$\begin{aligned} f(c,A,B,{\textbf{x}}) = f(c,B,A,\tilde{{\textbf{x}}}), \end{aligned}$$

where $\tilde{{\textbf{x}}}=(x_2,x_1)$ denotes the flipped concentration pair. That is, if the order of drugs and their concentrations were swapped, we should see the exact same viability on the cell line *c*. In fact, the dose–response surface of the pair (*A*, *B*) should be exactly the same as for the pair (*B*, *A*), but reflected around the 45$^\circ$ line, see Fig. 2. From Eq. (4), we note that this is easily achieved for the Bliss non-interaction assumption, $p_0({\textbf{x}})$, by simply swapping the arguments around. But in order to enforce the equality in Eq. (11), we need to encode the same structure into the joint model of the latent GPs across all experiments. If this symmetric relationship is not accounted for properly, the model can struggle to learn the inter-output correlations, and consequently negatively affect the performance of the model.

In a GP, these types of invariances and symmetries can be directly encoded in the kernel construction. This is achieved by writing the desired GP as a sum of terms in such a way that swapping arguments around yields an unchanged function [38]. In this case, we need to enforce the permutation invariance of the drug pairs and their concentrations.

Let

12 $$\begin{aligned} \begin{aligned} {\tilde{\zeta }}(c,A,B,{\textbf{x}}) \sim \mathcal{G}\mathcal{P}\left( 0,\kappa _{c}(c,c')\kappa _{d}((A,B),(A',B'))\kappa _{x}({\textbf{x}},{\textbf{x}}')\right) , \end{aligned} \end{aligned}$$

denote the multi-output GP with the product-structured covariance developed in the previous section. By writing the final evaluation at location $(c,A,B,{\textbf{x}})$ as a sum of the GP evaluated at two locations:

13 $$\begin{aligned} \zeta (c,A,B,{\textbf{x}}) = {\tilde{\zeta }}(c,A,B,{\textbf{x}}) + {\tilde{\zeta }}(c,B,A,\tilde{{\textbf{x}}}), \end{aligned}$$

we ensure that the function value is unchanged when the drugs and their concentrations switch places. That is, the mapping $(c,A,B,{\textbf{x}})\mapsto (c,B,A,\tilde{{\textbf{x}}})$ leaves the function unchanged. Since Gaussians are closed under addition, the GP prior on ${\tilde{\zeta }}$ induces a GP prior over $\zeta$, with mean zero and covariance function:

14 $$\begin{aligned} \begin{aligned} &\kappa _{\zeta }((c,A,B,{\textbf{x}}),(c',A',B',{\textbf{x}}'))= \kappa _c(c,c') \\&\quad \times \left[ \left( \kappa _d((A,B),(A',B')) + \kappa _d((B,A),(B',A'))\right) \kappa _x({\textbf{x}},{\textbf{x}}') \right. \\& \quad \left. + \left( \kappa _d((A,B),(B',A')) + \kappa _d((B,A),(A',B'))\right) \kappa _x(\tilde{{\textbf{x}}},{\textbf{x}}')\right] . \end{aligned} \end{aligned}$$

See the Additional file 1 for the derivation of this covariance function. Note that the kernel assumes that the two drug pairs (*A*, *B*) and (*B*, *A*) are distinct entities, hence $\kappa _d((A,B),(A',B')) \ne \kappa _d((B,A),(B',A'))$, and the kernel can not be simplified further. This assumption needs to be enforced when the drug combination kernel is constructed, otherwise the model will force all dose–response functions to be symmetric around the 45$^\circ$ line, instead of reflected only in the swapped drug pair (this point is expanded upon in the Additional file 1).

### Observation model

High-throughput drug combination screens produce measures of post-treatment cell viability given a range of different concentrations of drugs. For a single experiment, let ${\textbf{y}}=(y_1,\ldots ,y_n)$ denote the measured cell viability corresponding to drug concentrations ${\textbf{X}}=({\textbf{x}}_1,\ldots ,{\textbf{x}}_n)$. In a dose–response modelling setting these cell viabilities are assumed observed from the underlying dose–response function $f({\textbf{x}})$ subject to some added noise due to various biological and technical factors. For example, both [20] and [39] consider a heteroskedastic noise model,

15 $$\begin{aligned} y_i \sim {\mathcal{N}}(f({\textbf{x}}_i),\sigma _i^2), \ \ \sigma _i^2>0, \end{aligned}$$

and proceed in a fully Bayesian fashion to estimate parameters and various summary statistics of drug efficacy and interaction. The heteroskedastic structure arises from how cell viabilities are measured in-vitro and normalized. Viability is usually measured indirectly by comparing a marker associated with viability (e.g. ATP levels) to positive and negative controls. Due to the heterogeneity of cell growth, the variance of this marker is usually much higher in the negative controls than in the positive controls, i.e. there is typically much more variance in the viability estimate when all cells are still alive, compared to when all cells are dead. This translates into a heteroskedastic noise structure that varies across the dose–response surface.

#### A two-stage observation model

Ideally, we would want to directly utilize the dose response model from the “Bayesian dose–response model for single experiments” section, swapping out the latent single-output GP prior $z(\cdot )$ with the invariant multi-output GP prior $\zeta (\cdot )$ from the previous section. But because of the sample size of most drug combination screens, as well as the latent way the GP enters into the model formulation, a fully integrated workflow starting from the viability measurements is not computationally feasible to pursue. The latent GP formulation is not conjugate and would require expensive numerical approximations. We instead rely on an indirect way of predicting cell viability where the main quantities of interest are evaluations of the latent GP underlying each experiment.

Borrowing an idea from meta-analysis [40], we proceed in a two stage fashion. In the first stage, each experiment of the drug combination dataset is analysed separately to obtain estimates of the underlying latent single-output GP $z(\cdot )$. That is, given an input of cell viability and drug concentrations $({\textbf{y}}_{cAB},{\textbf{X}}_{cAB})$ from the experiment consisting of cell line *c*, and the drug combination (*A*, *B*), we use the Bayesian dose–response model from the “Bayesian dose–response model for single experiments” section, along with the heteroskedastic observation model in Eq. (15) to estimate the marginal posterior distribution of the latent GP at each drug concentration, i.e.

16 $$\begin{aligned} \pi \left( z({\textbf{x}}_{cAB,i}) \ \vert \ {\textbf{y}}_{cAB}, {\textbf{X}}_{cAB}\right) , \ i=1,\ldots ,n \end{aligned}$$

See the Additional file 1 for a full description of the model used to fit each experiment. From this posterior we compute the mean and variance, denoted ${\bar{z}}_{cAB,i}$ and $\sigma ^2_{cAB,i}$, and collect these in vectors, $\bar{{\textbf{z}}}_{cAB}=({\bar{z}}_{cAB,1},\ldots ,{\bar{z}}_{cAB,n})$ and $\varvec{\sigma }^2_{cAB}=(\sigma ^2_{cAB,1},\ldots ,\sigma ^2_{cAB,n})$. In this fashion the latent GP of each experiment is summarized by the vector $\bar{{\textbf{z}}}_{cAB}$ which will be used as the main response variable in the multi-output model. Furthermore, since each experiment contains different drugs and cell lines, and perhaps even utilizes different experimental designs or number of replicates, the estimation uncertainty will vary across the latent GP surface, and between different experiments. These differences in uncertainty are accounted for by the corresponding vector of posterior variance, $\varvec{\sigma }^2_{cAB}$, which will be used to implicitly weight observations by how precisely they are measured in the next stage.

In the second stage, the posterior mean estimates obtained in the first stage are considered as fixed observed quantities, noisily observed from the multi-output GP. Specifically, we assume Gaussian noise and write

17 $$\begin{aligned} \begin{aligned} {\bar{z}}_{cAB,i} \vert \ \zeta , {\textbf{x}}_i, y_i&= \zeta (c,A,B,{\textbf{x}}_i) + \varepsilon _{cAB,i}, \\ \zeta&\sim \mathcal{G}\mathcal{P}(0,\kappa _{\zeta }((c,A,B,{\textbf{x}}),(c',A',B',{\textbf{x}}')))\\ \varepsilon _{cAB,i} \vert \ {\textbf{x}}_i, y_i&\sim {\mathcal{N}}(0,\sigma ^2_{cAB,i}+\sigma ^2). \end{aligned} \end{aligned}$$

The error term is given a two-part variance, where the first term, $\sigma ^2_{cAB,i}$, is considered fixed and corresponds to the posterior variance estimated from the first stage. This term captures any measurement error of the latent GP corresponding to how the experiment was performed, e.g. number of replicates or experimental design. The second term, $\sigma ^2$ corresponds to a common error across all the outputs, which introduces a dependency across the outputs and helps to regularize the variance components. In regular multi-output GP regression, a common noise term is key to avoid issues of *autokrigability* [41], where the outputs become independent of each other in the noiseless setting. This parameter needs to be estimated from the data. Note that the model defined above is conditional on the measured cell viability $y_i$ only through the posterior distribution in Eq. (16). In order to keep notation to a minimum, we omit this dependence in the following sections, and simply consider $\bar{{\textbf{z}}}_{cAB}$ and $\varvec{\sigma }^2_{cAB}$ as fixed quantities.

### Kernel choices

Having set up the general structure of the model we now discuss the specific choices for the input, drug combination and cell line kernels that are used to produce the results in this paper.

*The input kernel* For the kernel controlling the function’s behaviour across the inputs, or drug concentrations ${\textbf{x}}$, we use a simple squared exponential kernel

18 $$\begin{aligned} \kappa _{x}({\textbf{x}},{\textbf{x}}') = \exp \left( -\frac{\vert \vert {\textbf{x}}-{\textbf{x}}'\vert \vert ^2}{2\ell ^2}\right) , \end{aligned}$$

where $\ell$ denotes the lengthscale parameter, controlling how sensitive the function is to changes in the input. The squared exponential kernel produces functions that vary smoothly with its covariates. It reflects the assumption that a small change in the drug concentrations produces a similarly small change in the output. Note that when fitting cell viability measurements of individual experiments to compute posterior means and variances of the latent GP in the “Bayesian dose–response model for single experiments” section, we utilize the default Matérn kernel recommended in [20] as a less smooth alternative to the squared exponential. This enables the posterior distribution to better adapt to rapid changes in the interaction surface, as well as give a more faithful representation of the underlying measurement error. When working in a multi-output setting using posterior means of each experiments’ latent GP as a response variable we prefer the smoother squared exponential kernel. The added smoothness helps to regularize the model and better estimate the covariance between outputs.

Given a set of unique drug concentrations ${\textbf{X}}=({\textbf{x}}_1,\ldots ,{\textbf{x}}_n)$ the Gram matrix $K_x$ generated by the kernel has entries $\{K_x\}_{ij}=\kappa _x({\textbf{x}}_i,{\textbf{x}}_j)$. If we assume further that the *n* unique drug concentrations lie on the two-dimensional Cartesian grid generated by a set of common drug concentrations, e.g. ${\textbf{X}}=X \times X$, where $X=(x_1,\ldots ,x_k)$, then the matrix generated by the ’reflected’ function $\kappa _x(\tilde{{\textbf{x}}},{\textbf{x}}')$ in Eq. (14) is simply a row permutation of $K_x$. That it, there exists a symmetric permutation matrix ${\tilde{P}}$ such that $\{{\tilde{P}}K_x\}_{ij}=\kappa _x(\tilde{{\textbf{x}}},{\textbf{x}})$. To see why this is true, note that the *i*th row of $K_x$ contains the entries $\kappa _x({\textbf{x}}_i,{\textbf{x}}_j)$ for $j=1,\ldots ,n$. Because ${\textbf{X}}$ is generated from a common set of concentrations, the reflected version of ${\textbf{x}}_i$ is also a valid concentration on the grid, and hence $\tilde{{\textbf{x}}}_i={\textbf{x}}_{i'}$ for some index $i'$. This holds for all of the *n* unique concentrations, and thus the matrix generated by $\kappa _x(\tilde{{\textbf{x}}},{\textbf{x}}')$ is simply a row-permutation of $K_x$.

*The drug combination kernel* A kernel over drug combinations can be constructed in many different ways, and sources of auxiliary information exists both for the individual compounds (their targets, mechanism of action, or chemical structure), and additionally for properties of the combinations themselves that are not reducible to the individual compounds. For example, if the two drugs are jointly targeting a synthetically lethal gene pair [42] that induces cell death, this property cannot be reduced back to the targets of the individual drugs.

The simplest method for constructing a drug combination kernel would be to simply combine together two single-drug kernels by their product. If (*A*, *B*) and $(A',B')$ denote two drug combinations, the kernel between these two could be written as

19 $$\begin{aligned} \kappa _{\text{d}}((A,B),(A',B')) = \kappa _{\text{dA}}(A,A')\kappa _{\text{dB}}(B,B'), \end{aligned}$$

where $\kappa _{\text{dA}}$ denotes the kernel function for drugs in the first position of a pair, and similarly $\kappa _{\text{dB}}$ for drugs in the second position. This construction is simple, and very computationally efficient due to the induced Kronecker structure of the covariance matrix. However, it is very limited in the type of information it can encode, being essentially restricted to only using information regarding the individual compounds. The Kronecker structure essentially encodes an independence assumption between the two drugs in a pair, precisely the opposite of what drug interaction is meant to capture. For this reason, such a simple construction is unsuitable for our purpose.

We instead opt for treating each drug combination as its own distinct entity, irreducible to its constituent parts, which allows for a much richer covariance structure, but is computationally more expensive. For a given dataset, let $N_d$ denote the number of unique drug pairs, which we assume can be ordered alphabetically, e.g. as

20 $$\left[ (A,B),(A,C),\ldots ,(A,Z),(B,C),\ldots , (B,Z),\ldots ,(W,Z)\right] .$$

We then extend this vector to include all the swapped pairs to obtain the ordering

21 $$\begin{aligned} \begin{aligned} \left[ (A,B),(A,C),\ldots ,(A,Z),(B,C),\ldots ,(B,Z),\ldots ,(W,Z), \right. \\ \left. (B,A),(C,A),\ldots ,(Z,A),(C,B),\ldots ,(Z,B),\ldots ,(Z,W) \right] . \end{aligned} \end{aligned}$$

Using this extended vector of drug combinations, the $2N_d \times 2N_d$ covariance matrix of drug pairs could be built in many ways. In this paper, we follow [41] and construct a “free-form” low-rank kernel matrix

22 $$\begin{aligned} K_d = L_dL_d^T + \text{diag}(\mathbf {v_d}), \ \ L_d \in {\mathbb {R}}^{2N_d\times r_d}, \ \mathbf {v_d}\in {\mathbb {R}}^{2N_d} \end{aligned}$$

where $r_d$ denotes the rank of the final matrix, and the entries of $L_d$ and $\mathbf {v_d}$ are considered parameters to be learned from the data. The rank of the matrix, $r_d$ is treated as a user-defined hyperparameter, and requires tuning.

The low-rank assumption can make a lot of sense in this setting, as most drug combination are non-interactive, typically only a few within a dataset show large deviations from the non-interaction model. The “free-form” structure of $K_d$, where the entries of the matrix are estimated from the data directly, allow for very complex covariance structures to be fit, but will not be able to extrapolate beyond the training data. That is, since the kernel does not have a parametric form that incorporates auxiliary data about the drug combinations, it is impossible to use this kernel to predict dose–response for combinations not already in the training dataset. For the purpose of this paper this restriction is fine, but we outline in the conclusion possible extensions that would allow the model to predict on unseen drugs or drug combinations.

This covariance matrix in Eq. (22) corresponds to the kernel function $\kappa _d((A,B),(A',B'))$ in Eq. (14), where the drug pairs are ordered alphabetically according to the ordering in (21). The other covariance matrices corresponding to the swapped drug pairs can be derived by permuting the rows and columns of $K_d$. Specifically, let *P* denote the $2N_d \times 2N_d$ symmetric permutation matrix of the form

$$\begin{aligned} P=\begin{bmatrix}{\textbf{0}} &{}\quad {\textbf{I}}_{N_d} \\ {\textbf{I}}_{N_d} &{}\quad {\textbf{0}}\end{bmatrix}, \end{aligned}$$

where ${\textbf{I}}_{N_d}$ denotes the identity matrix of size $N_d$. It is easy to check that the matrices $PK_dP$, $K_dP$ and $PK_d$ are the matrices generated by the functions $\kappa _d((B,A),(B',A'))$, $\kappa _d((A,B),(B',A'))$, and $\kappa _d((B,A),(A',B'))$, respectively.

*Cell line kernel* For the cell line kernel, we use the same low-rank structure as for the drug combination kernel. That is, given a dataset with $N_c$ distinct cell lines, we create a low-rank kernel matrix by

23 $$\begin{aligned} K_c = L_cL_c^T + \text{diag}(\mathbf {v_c}), \ \ L_c \in {\mathbb {R}}^{N_c\times r_c}, \ \mathbf {v_c}\in {\mathbb {R}}^{N_c}, \end{aligned}$$

where again $r_c$ denotes the rank of the final matrix and must be tuned, and the entries of $L_c$ and ${\textbf{v}}_d$ are considered parameters to be learned from the data.

### Inference and parameter learning

In this section, we describe the general form of the *predictive posterior distribution*, $\pi (\zeta (c,A,B,{\textbf{x}}_*) \vert \ {\textbf{Z}},{\textbf{S}},{\textbf{X}})$, of the GP evaluated at a new drug concentration ${\textbf{x}}_*$ in the experiment consisting of cell line *c*, and drug combination (*A*, *B*). We also describe an efficient procedure for estimating the kernel parameters by maximizing the marginal likelihood.

We start by introducing some rather strict assumptions regarding the data setup, that we later relax to a more realistic setting suitable for drug combination datasets. The first restriction we will make is that each experiment in the training dataset has been performed on a common set of drug concentrations, ${\textbf{X}}=({\textbf{x}}_1,\ldots ,{\textbf{x}}_n)$. Additionally, we demand that each of these *n* unique drug concentrations lie on the two-dimensional Cartesian grid generated by some set of common concentrations, i.e. ${\textbf{X}}=X\times X$, where $X=(x_1,\ldots ,x_k)$ and thus $n=k^2$. Secondly, we require that every drug combination has been evaluated on every cell line, i.e. that the data is complete. Note that since we insisted in the definition of the invariant kernel in Eq. (14), that the combinations (*A*, *B*) and (*B*, *A*) are distinct entities, this entail that that we need two versions of every experiment; one for each ordering of the pair. In practice, there is no natural ordering of the drugs, and no way to differentiate between the two orderings in an experimental setting. We show later that there is no need to enter the data twice, we simply treat the unobserved ordering as missing data when doing inference.

Given a complete drug combination dataset consisting of $N_d$ drug combinations, each screened on $N_c$ cell lines at *n* distinct drug concentrations on the Cartesian grid as defined above. Let ${\textbf{Z}}$ denote the $n\times (N_c \cdot 2N_d)$ matrix containing posterior means of the latent GP from each experiment, including both orderings of the initial $N_d$ drug combinations. The columns of ${\textbf{Z}}$ are ordered such that the first $2N_d$ columns correspond to the drug combinations in the first cell line, the next $2N_d$ to the second cell line and so on. Let ${\textbf{S}}$ denote the matrix of the same dimensions, containing the corresponding posterior variances. Then, given the model structure in (17), the predictive posterior distribution of the GP at an unobserved input ${\textbf{x}}_*$, in the experiment corresponding to the cell line *c* and the drug combination (*A*, *B*), conditional on the data $({\textbf{Z}},{\textbf{S}})$ is available in analytic form [43]

24 $$\begin{aligned} \begin{aligned} \zeta (c,A,B,{\textbf{x}}_*) \vert \ {\textbf{Z}}, {\textbf{S}}&\sim {\mathcal{N}}(\bar{\zeta _*},\text{V}[\bar{\zeta _*}]), \\ \bar{\zeta _*}&= \mathbf {k_*}^T(K+\Sigma )^{-1}\text{vec}({\textbf{Z}}), \\ \text{V}[\bar{\zeta _*}]&= k_{**}-\mathbf {k_*}^T(K+\Sigma )^{-1}\mathbf {k_*}, \\ \Sigma&= \text{diag}(\text{vec}({\textbf{S}})+\sigma ^2) \end{aligned} \end{aligned}$$

where $$k_{**}=\kappa _{\zeta }((c,A,B,{\textbf{x}}_*),(c,A,B,{\textbf{x}}_*))$$. The vector $${\textbf{k}}_*$$ contains the covariances between the test point $$(c,A,B,{\textbf{x}}_*)$$ and the training data, and can be written as $${\textbf{k}}_*=(K_c\otimes K_d)^{cAB}\otimes {\textbf{k}}_*^x$$, where $$(K_c\otimes K_d)^{cAB}$$ selects the column of $$K_c \otimes K_d$$ corresponding to the cell line *c* and drug combination (*A*, *B*), and $${\textbf{k}}_*^x$$ denotes the covariance between the new drug concentration $${\textbf{x}}_*$$ and the concentrations in the training data. Note that all outputs can be predicted simultaneously by replacing the vector $${\textbf{k}}_*$$ with the matrix $$K_*=(K_c\otimes K_d) \otimes {\textbf{k}}_*^x$$, using the full matrix $$K_c \otimes K_d$$ instead of only selecting the column corresponding the desired experiment.

The prior covariance matrix *K* takes a particularly simple form in this fully observed setting, and can be written as

25 $$\begin{aligned} K = K_c\otimes \left[ \left( K_d + PK_dP\right) \otimes K_x + \left( PK_d + K_dP\right) \otimes {\tilde{P}}K_x \right] , \end{aligned}$$

where $K_c$ is the $N_c \times N_c$ matrix of cell line covariances, $K_d$ the $2N_d \times 2N_d$ matrix of drug combination covariances, and $K_x$ the matrix of drug concentration covariance. The matrices *P* and ${\tilde{P}}$ are symmetric permutation matrices, binary matrices with a single 1 entry in each row and column. These matrices permute the rows or columns of a matrix *D* when either pre- (*PD*) or post-multiplying (*DP*).

The predictive distribution above is for fixed values of the kernel parameters $\{\ell ,L_d,{\textbf{v}}_d,L_c,{\textbf{v}}_c\}$ and the global noise parameter $\sigma ^2$. These parameters can be learned from the data following the standard GP practice of maximizing the marginal likelihood. Since the model is conjugate, the GP can be integrated out to obtain the likelihood of the data conditioned on the model parameters, $\varvec{\theta }=\{\ell ,L_d,{\textbf{v}}_d,L_c,{\textbf{v}}_c\,\sigma^2\}$ [43], and can be written down in closed form. For the fully observed dataset described above, the log marginal likelihood can be written as

26 $$\begin{aligned} \begin{aligned} \text{log}({\textbf{Z}} | {\textbf{S}},{\textbf{X}},\varvec{\theta }) = \text{const.} - \left[ \text{vec}({\textbf{Z}})^T\left( K+\Sigma \right) ^{-1}\text{vec}({\textbf{Z}}) \ + \log \vert K+\Sigma \vert \right] , \end{aligned} \end{aligned}$$

where the $\text{vec}({\textbf{Z}})^T\left( K+\Sigma \right) ^{-1}\text{vec}({\textbf{Z}})$ can be thought of as measuring the model’s fit to the data, and the log-determinant $\log \vert K+\Sigma \vert$ penalizing model complexity. This log marginal likelihood can be maximized using any gradient based optimizer.

There are two main issues with the expressions in Eqs. (24) and (26). First, note that the inverse product $(K+\Sigma )^{-1}\text{vec}({\textbf{Z}})$ appears in both the predictive posterior distribution, and in the marginal likelihood. The standard way of computing this term is through a Cholesky decomposition of $K+\Sigma$, from which also the log-determinant is readily available. However, the Cholesky decomposition requires ${\mathcal{O}}(N^3)$ computations [43], where *N* is the number of total observations, i.e. the number of entries in ${\textbf{Z}}$. For most drug combination datasets, with number of observations in the hundreds of thousands, this is simply not computationally feasible. The second issue is that the predictive posterior distribution in Eq. (24) is of limited use. Since each drug combination has been observed on each cell line, there is nothing left to predict besides the value of the latent GP at unobserved concentration points ${\textbf{x}}_*$. Both of these issues are solved in the next section, where we relax the assumptions on the data setup to incomplete settings, and provide methods for computing the predictive posterior distribution and the marginal likelihood using incomplete data.

#### Incomplete data

In recent years, a lot of work has been put into making GPs more scalable [44], either by keeping inference exact and speeding up the calculations in Eqs. (24) and (26), or by various methods of approximate inference, such as variational approaches. In this paper, we make use of the highly structured covariance matrix to keep inference exact, but resort to a minor approximation for of the log-determinant used in parameter estimation. More specifically, we rely on the structure of the covariance matrix *K* to drastically speed up these computations by first exploiting fast matrix–vector multiplications for Kronecker matrices, and secondly by proving a novel result regarding the eigenvalues of *K* that entails that the log-determinant can be computed efficiently.

For Kronecker-structured covariance matrices, i.e. those that can be written as $K=K_1\otimes \cdots \otimes K_d$, [45] developed fast inference procedures in the fully observed setting and under homoskedastic noise. By exploiting properties of the Kronecker product, both the log-determinant and the inverse can be computed efficiently, resulting in a method that requires only ${\mathcal{O}}(N)$ computations. This procedure was later extended by [46] to allow fast Kronecker inference also when the covariance matrix is not necessarily of this form. The main idea is to first generate dummy observations such that the covariance matrix of the joint dataset can be written as a Kronecker product. Then, these dummy observations are effectively ignored during inference by adding a large corresponding noise term, and computing the inverses using conjugate gradients (CG). While the covariance matrix *K* in Eq. (25) is not completely Kronecker-structured because of the matrix sums, we can still apply this trick since CG only relies on efficient matrix–vector multiplication.

We utilize this trick to lift the initial restrictions made on the training dataset. Given a partially observed drug combination dataset of size *M*, i.e. not every drug combination is screened on every cell line, and not every experiment is performed using the exact same grid of concentrations. Let $K_M$ denote the kernel matrix generated by (14), which in this partially observed case will not necessarily have any special structure, and let $\Sigma _M$ denote the corresponding diagonal noise matrix. Let further ${\textbf{z}}$ denote the vector of length *M*, containing the estimated posterior means $\bar{{\textbf{z}}}_{cAB}$ of the latent GP underlying each experiment.

When constructing the matrix ${\textbf{Z}}$ corresponding to the fully observed dataset, we utilize the values from ${\textbf{z}}$ wherever possible, and fill in unobserved entries using dummy values, $z_0$. Then, for the corresponding entry in ${\textbf{S}}$ we add a large fixed noise, e.g. $\epsilon ^{-1}$ for a sufficiently small $\epsilon >0$. For example, if the experiments in the screen have all been performed at slightly different concentrations, we construct a common grid across all experiments and add dummy observations at whichever points are missing in each experiment.

We then used preconditioned CG to compute $(K+\Sigma )^{-1}\text{vec}({\textbf{Z}})$, using $C=\Sigma ^{-1/2}$ as a preconditioner matrix to solve the linear system $C^T(K+\Sigma )C{\textbf{q}}=C^T\text{vec}({\textbf{Z}})$. As shown in [46], this procedure effectively ignores the contributions of the dummy observations, and produces inference identical to the naïve implementation. That is, solving the pre-conditioned system above is exactly equal to solving $(K_M + \Sigma _M){\textbf{q}}={\textbf{z}}$, only using the observed values.

In order to learn the parameters of the model, we still need to compute the log-determinant in Eq. (26). For this term, we utilize the same approximation as in [46]:

27 $$\begin{aligned} \log \vert K_M + \Sigma _M \vert =\sum _{i=1}^M \log \lambda _i^M \approx \sum _{i=1}^M \log {\tilde{\lambda }}_i, \end{aligned}$$

where the eigenvalues $\lambda _i^M$ of $(K_M+\Sigma _M)$ are approximated by the eigenvalues $\lambda _i$ of $(K+\Sigma )$ such that ${\tilde{\lambda }}_i=\frac{M}{N}\lambda _i$. In order to obtain the eigenvalues of the sum $K+\Sigma$, we first use Weyl’s inequality [47] to upper bound each $\lambda _i$ by the eigenvalues of *K* and $\Sigma$. Let $\lambda ^K_1 \le \cdots \le \lambda ^K_N$ and $\lambda ^\Sigma _1 \le \cdots \le \lambda ^\Sigma _N$ denote the sorted eigenvalues of the matrices *K* and $\Sigma$, respectively. Then, by Weyl’s inequality we have

28 $$\begin{aligned} \lambda ^{K+\Sigma }_{i+j-1} \le \lambda ^K_i + \lambda ^\Sigma _j, \ \text{for all } i, j, \end{aligned}$$

with some freedom in how the indices $\{i,j\}$ are chosen. Following [48], we adopt the heuristic of setting $i=j$ whenever possible, and opt for $j=i+1$ otherwise. The eigenvalues of $\Sigma$ are readily available since it is a diagonal matrix, but we are still left with computing the eigenvalues of *K*, an often prohibitively large matrix. Initially, it looks like this will be difficult due to the matrix sums disrupting any natural Kronecker structure we could exploit. However, it turns out that due to the symmetries of this matrix, we can indeed exploit Kronecker algebra to efficiently get the eigenvalues of *K*. The following proposition is proved in the Additional file 1.

##### Proposition 1

Let $K_c$, $K_d$ and $K_x$ denote positive semi-definite matrices, while *P* and ${\tilde{P}}$ denote symmetric permutation matrices. Furthermore, let $\sigma (A)^+$ denote the collection of positive eigenvalues of the matrix *A*. Then, the matrix

$$\begin{aligned} K = K_c\otimes \left[ \left( K_d + PK_dP\right) \otimes K_x + \left( PK_d + K_dP\right) \otimes {\tilde{P}}K_x \right] , \end{aligned}$$

is positive semi-definite (PSD) and

$$\begin{aligned} \sigma (K)^+ = 2\sigma (K_c \otimes \left( PK_d + K_dP\right) \otimes {\tilde{P}}K_x)^+ \end{aligned}$$

Thus, all the eigenvalues of *K* can be computed from the positive eigenvalues of the Kronecker-structured matrix $K_c \otimes \left( PK_d + K_dP\right) \otimes {\tilde{P}}K_x$. This can be performed efficiently since the eigenvalues of a Kronecker-structured matrix is simply the cross-product of the eigenvalues from each individual matrix.

Using these two tricks: pre-conditioned CG for the inverse solve and the approximation for the log-determinant, inference and parameter learning can be done efficiently and scales to large and incompletely observed datasets. The predictive posterior distribution in Eq. (24) is now of more use, since it can be used to predict the values of the latent GP in experiments that have not been run. In the next section we show how these predictions can be used to reconstruct the full dose–response function. The bottle-neck in these computations will usually be the eigendecomposition of $PK_d+K_dP$, typically the largest of the three matrices utilized to create *K*. The model is implemented using in GPyTorch [31], a library for GP inference built on top of the efficient PyTorch machine learning framework [49]. We use the built-in functionality of automatic differentiation to compute the requires gradients of the marginal likelihood and rely on GPyTorch’s implementation of preconditioned conjugate gradients.

### Predicting dose–response and drug interaction

The inference procedure described in the preceding sections produces estimates of the multi-output GP $\zeta (\cdot )$ evaluated at an unobserved output $(c,A,B,{\textbf{x}}_*)$. In order to obtain a prediction of dose response at this location, we plug these estimates into the dose response model of “Bayesian dose–response model for single experiments” section. First, we use the mean of the predictive distribution in (24), ${\bar{\zeta }}*$, to obtain an estimate of the interaction effect at this point, ${\hat{\Delta }}_*$.

29 $$\begin{aligned} \begin{aligned} {\hat{\Delta }}_* = {\hat{g}}({\bar{\zeta }}_*) \\ {\hat{g}}({\bar{\zeta }}_*) = \frac{-{\hat{p}}_{0*}}{1+\exp \left[ b_1{\bar{\zeta }}_*+\log \left( \frac{{\hat{p}}_{0*}}{1-{\hat{p}}_{0*}}\right) \right] } \\ \quad +\frac{1-{\hat{p}}_{0*}}{1+\exp \left[ -b_2{\bar{\zeta }}_*-\log \left( \frac{{\hat{p}}_{0*}}{1-{\hat{p}}_{0*}}\right) \right] }, \end{aligned} \end{aligned}$$

where ${\hat{p}}_{0*}$ denotes the evaluation of ${\hat{p}}_{0}({\textbf{x}}_*)$, which has been estimated using the monotherapy data from drugs *A* and *B* on cell line *c*, but no combination data (see the Additional file 1). For the parameters $(b_1,b_2)$, which cannot be estimated without access to the combination data, we use the posterior means of these parameters as estimated from the experiments in the training data.

Thus, we can obtain an estimate of the dose–response at an unobserved input, ${\hat{f}}_*=f(c,A,B,{\textbf{x}}_*)$ simply adding the two terms together:

30 $$\begin{aligned} {\hat{f}}_* = {\hat{p}}_{0*} + {\hat{\Delta }}_*. \end{aligned}$$

By doing this for a range of drug concentrations ${\textbf{X}}_*=({\textbf{x}}_{1*},\ldots ,{\textbf{x}}_{n*})$, the whole dose–response surface can be reconstructed across a grid of concentrations.

## Data and preprocessing

To test the predictive performance of our method, we utilize the publicly available dataset provided by [10]. In this dataset, 38 drugs were combined in a pairwise manner into 583 distinct combinations that were screened on 39 cancer cell lines across 6 different tissues of origin (Lung, 8; Ovarian, 9; Melanoma, 6; Colon, 8; Breast, 6; Prostate, 2). Each combination was screened on all 39 cell lines, providing a total of 22,737 drug combination experiments. Within each experiment, the drugs are combined on a $4\times 4$ grid of concentrations, and at each location cell viability is measured in 4 replicates. Additionally for each single drug response, cell viability is measured for at least 8 distinct drug concentrations, with replicates varying from three to six. In total, the dataset consists of over 1.5 million cell viability measurements.

### Pre-processing

For pre-processing, we ran each experiment through the bayesynergy [20] R package, to individually fit each experiment to the dose–response model in the “Bayesian dose–response model for single experiments” section. We utilized version 2.4.1 of the package on default settings, resulting in 4000 samples from the posterior distribution of each individual experiment. Since the experiments are performed on different grids of concentrations, we scaled the concentration ranges of each experiment to the $[0,1]\times [0,1]$ unit box and constructed a common $10\times 10$ grid of concentrations in an equispaced manner across the [0, 1] range. We then sampled the corresponding latent GP evaluated at these locations from the posterior predictive distribution, and computed means and variances at these locations. This results in a fully observed dataset on this common grid of drug concentrations. The scaling to the unit box is not strictly necessary for the inference procedure described in the “Inference and parameter learning” section to be applied, we could construct a complete grid starting from the original concentrations. But because the concentration ranges of individual drugs can be very different from each other, the scaling to a common range makes it easier to fit the data using the same length-scale $\ell$ in the input kernel across all outputs.

We construct the $({\textbf{Z}},{\textbf{S}})$ matrices by following the alphabetical ordering established previously. Thus in the first $2N_d$ columns, which corresponds to the first cell line in an alphabetical ordering, the first half of the columns contain data, while the second half, correponding to the alphabetically reversed ordering of the drug combination, is treated as missing data. We set the missing entries in ${\textbf{Z}}$ equal to the value $z_0=-999$ and further set the corresponding entries of ${\textbf{S}}$ equal to $\epsilon ^{-1}$ where $\epsilon =1e^{-12}$.

Additionally, from the output of the bayesynergy package for each individual experiment, we also generate samples from the posterior predictive distribution of the dose–response function $f({\textbf{x}})$, evaluated at the complete grid of concentrations. From these samples, we compute posterior means and variances, and collect them in matrices ${\textbf{F}}$ and ${\textbf{S}}_F$ using the same ordering as for ${\textbf{Z}}$ and ${\textbf{S}}$.

## Prediction setting, performance metrics and results

We evaluate the prediction performance of the model in completely held out, untested experiments. By an experiment, we mean a (cell line, drug A, drug B) triplet. In this setting, we assume that the training dataset contains at least some previous experiments for each cell line and drug combination of interest, but that the particular experiment we wish to predict has not been performed.

Given the fully observed dataset, we hold out entire experiments from the training dataset by treating the corresponding columns of ${\textbf{Z}}$ as missing data. We then predict these missing columns using the predictive posterior distribution in Eq. (24), and construct the corresponding dose–response function for that experiment (“Predicting dose–response and drug interaction”). For the test dataset we use the same grid of drug concentrations as for the training dataset, and we predict at every output simultaneously. In this setting, the covariance between the test and training inputs (the vector ${\textbf{k}}_*$ in Eq. 24) is simply the kernel matrix *K* and we can write

31 $$\begin{aligned} \begin{aligned} \widehat{\text{vec}({\textbf{Z}})}=K^T(K+\Sigma )^{-1}\text{vec}({\textbf{Z}}), \end{aligned} \end{aligned}$$

for the predictive mean. In a way, the prediction procedure can be thought of as imputing the missing entries of ${\textbf{Z}}$. These predictions can then be used to construct estimated of the dose–response function ${\hat{f}}_*$ of untested experiments, by following the procedure in the “Predicting dose–response and drug interaction” section.

### Parameter tuning via cross-validation

We divide the full dataset using an 80/20 train/test split, by randomly sampling the experiments. We retain 4,547 experiments for testing and 18,190 for training. There are two hyper-parameters in the model that need tuning, $(r_c,r_d)$ controlling the rank of the cell line and drug combination kernels. To select these, we perform 5-fold cross validation using the training dataset, across a range of values: $r_c\in \{1,3,5,10,20\}$ and $r_d\in \{10,100,200,300,600\}$. These ranges can be chosen as e.g. percentiles of the total number of drugs and cell lines in the dataset, while still maintaining a sense of “low” rank. This encodes an assumption that the cell lines and drugs can be effectively summarised by a lower-dimensional representation. Note that for the drug combination rank we set the rank according to the full matrix of drug combinations, in both orderings. For this dataset, $K_d$ has dimensions $1166\times 1166$. After the optimal parameters have been chosen, the model is validated on the test set.

### Performance metrics

The primary metric utilized to evaluate performance in the cross-validation is the weighted root-mean-squared-error (wRMSE) of the latent GP predictions. Since each observation in the test set has a corresponding measurement uncertainty, we weight observations higher if they are measured precisely. This is done simply by weighting with the inverse measurement uncertainty. For the latent GP, the wRMSE can be written as,

32 $$\begin{aligned} \begin{aligned} \text{wRMSE}=\sqrt{\sum _{i\in {\mathcal{T}}}w_i\left( \widehat{\text{vec}({\textbf{Z}})}_i-\text{vec}({\textbf{Z}})_i\right) ^2},\\ w_i= \frac{{\tilde{w}}_i}{\sum _{i\in {\mathcal{T}}}{\tilde{w}}_i}, \ \text{where } {\tilde{w}}_i = 1/\text{vec}({\textbf{S}})_i \end{aligned} \end{aligned}$$

where the sums are taken over the indices corresponding to the test set ${\mathcal{T}}$. We additionally compute the Pearson correlation coefficient between predicted and observed as a secondary metric.

We utilize the same metrics to evaluate the performance of dose–response prediction, where the predicted dose–response is computed from the latent GP using the procedure in the “Predicting dose–response and drug interaction” section). Let $\widehat{\text{vec}({\textbf{F}})}$ denote the vector of predicted dose–response, generated by a pushing each entry of $\widehat{\text{vec}({\textbf{Z}})}$ through Eq. (30). Then the wRMSE for dose–response prediction is,

33 $$\begin{aligned} \begin{aligned} \text{wRMSE}=\sqrt{\sum _{i\in {\mathcal{T}}}w_i\left( \widehat{\text{vec}({\textbf{F}})}_i-\text{vec}({\textbf{F}})_i\right) ^2},\\ w_i= \frac{{\tilde{w}}_i}{\sum _{i\in {\mathcal{T}}}{\tilde{w}}_i}, \ \text{where } {\tilde{w}}_i = 1/\text{vec}(\mathbf {S_F})_i, \end{aligned} \end{aligned}$$

where again the sums are taken over the indices corresponding to the test set.

### Comparison models

As a baseline of comparison for the model’s ability to predict dose–response, we utilize the non-interaction model ${\hat{p}}_{0*}$., i.e. the Bliss model where the joint effect is assumed to be the product of the two monotherapy curves. In our modelling, we essentially predict deviations from the Bliss model, and hence it is a natural model to compare against. Additionally, in our experience large deviations from the Bliss model are rare, and most experiments are fit well by such a simple model. We compute ${\hat{p}}_{0*}$ using the bayesynergy R package, utilizing only monotherapy data.

We also compare our framework against a state-of-the-art model for this prediction problem, the comboLTR model [24]. comboLTR makes use of a higher-order polynomial regression model, including all interaction terms to predict dose–response in unseen experiments. The model takes as input raw viability measurements from each experiment, alongside labels of the cell lines, drugs and drug concentrations, and can further make use of various auxiliary information sources regarding the drugs or the cell lines. Following the recommendations in the paper, we set the order of the polynomial to five, and use one-hot encoding to represent the cell lines, drugs and drug concentrations. We tune the hyperparameters of the model using the same train/test split and cross-validation folds of experiments as for the PIICM. In order to compare the predictions made by comboLTR (which is trained using the original concentrations), with those made by PIICM (which scales all experiments to a common grid on the unit box), we use the trained PIICM to predict on a new grid of observations via the standard predictive posterior distribution in Eq. (24). For each experiment, the grid is selected such that it corresponds to the original concentrations that were used to measure viability. Hence, we can compare both PIICM’s and comboLTR’s performance in predicting raw viability measurements. Since we do not have a measure of uncertainty for the raw viability measurements, we use the regular RMSE as a primary metric and Pearson correlation as secondary metric. In the Additional file 1 we also provide a short discussion and comparison of our model with the IDACombo framework [25], which can be viewed as an alternative baseline $p_0$ for comparison since it relies solely on monotherapy measurements.

### Parameter initialization and optimization

The optimization of the marginal likelihood was performed with the Adam optimizer [50], using a learning rate of 0.1 for all parameters, and training until no more improvement was seen in the marginal likelihood.

The parameters of the model that are constrained to be positive were initialized as follows:

34 $$\begin{aligned} \begin{aligned} \ell , \sigma = \log \left( 1+\exp (0)\right) \approx 0.693 \\ v_{c,i} = \log \left( 1+\exp ({\tilde{v}}_{c,i})\right) , \ {\tilde{v}}_{c,i} \sim {\mathcal{N}}(0,1), \\ \text{for } {\textbf{v}}_c=(v_{c,1},\ldots ,v_{c,N_d}) \\ v_{d,i} = \log \left( 1+\exp ({\tilde{v}}_{d,i})\right) , \ {\tilde{v}}_{d,i} \sim {\mathcal{N}}(0,1), \\ \text{for } {\textbf{v}}_d=(v_{d,1},\ldots ,v_{d,2N_d}), \end{aligned} \end{aligned}$$

while the unconstrained entries of the matrices $L_c$ and $L_d$ were each drawn independently from a normal distribution with standard deviation of 0.1. Note that this has the effect that the covariance matrices $K_d$ and $K_c$ are initialized close to a diagonal matrix.

All CG solves were done to a tolerance of $0.1\alpha$, where $\alpha =M/N$ is the proportion of observed entries in the matrix ${\textbf{Z}}$. This was done to ensure that each setting was evaluated to the same level of precision. We utilized a high performance cluster to run the model, taking advantage of an NVIDIA P100 GPU with 16GB of memory to speed up the required matrix–vector multiplications of CG. A single run of the model, including model training and prediction took around 30 min to complete using this setup.

### Results

**Fig. 3:**
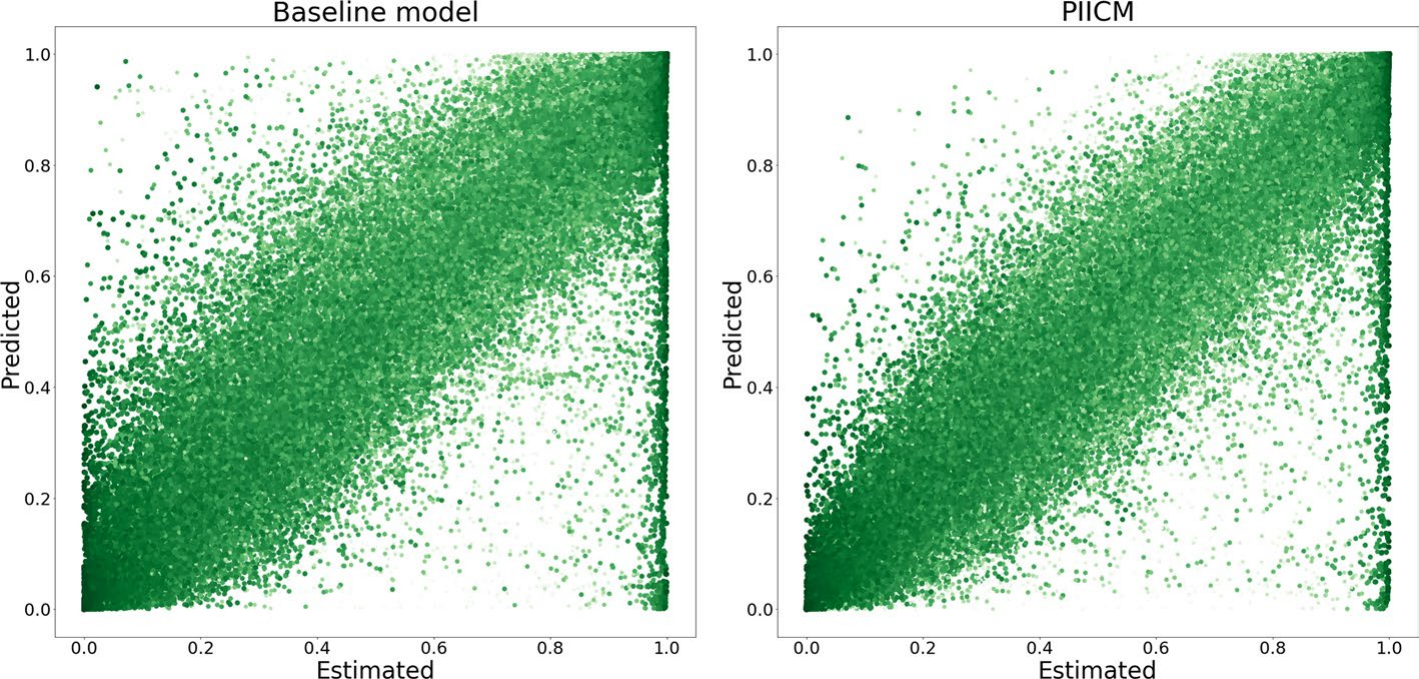
The figure shows estimated versus predicted dose–response for both the baseline model (left panel) and the PIICM (right panel). The estimated dose–response values correspond to the posterior mean of the dose–response function, estimated for each experiment using the bayesynergy R package, and collected in the matrix ${\textbf{F}}$. The plot is colored according to the corresponding measurement error, darker color indicating more certainty regarding the estimate. Note the improvement from baseline, particularly in points in the upper-left corner

The results from the cross-validation are shown in the first line of Table 1, with both the primary (wRMSE) and secondary (Pearson correlation) metric of interest alongside the optimal ranks selected. Additionally, we compute the same metrics for the dose–response predictions computed using the procedure in  the “Predicting dose–response and drug interaction” section, and report these alongside the baseline performance. For dose–response prediction, the results of the baseline model are already quite good, with our model only showing a small improvement (Pearson correlation of 0.98 compared to 0.97, and wRMSE of 0.068 compared to 0.0724). The optimal ranks selected are $r_d=200$ and $r_c=10$, indicating that the model is finding some structure in the training data. These results are also visualized in Fig. 3, where we plot estimated versus predicted dose–response for PIICM (right panel) and baseline (left panel). Each point in the figure corresponds to an evaluation of the dose–response function *f* for a given (cell line, drug A, drug B, conc. drug A, conc. drug B) quintuplet. The x-axis corresponds to the dose–response as estimated from the raw viability measurements of each experiment using the bayesynergy R package, while the y-axis corresponds to the predicted values. By comparing the left and right panel, we can see that the improvements from the baseline are mainly observed in the top-left corner of the plot. In this region, the predicted value is close to one, indicating near 100% cell viability, but the estimated values are near zero. For the baseline model (left panel), this area represents points of large synergistic effects, as the Bliss non-interaction is predicting values near one, while the true estimates are near zero. The predictions made by PIICM (right panel), have fewer points in this region, which gives some indication that the model is able to capture synergistic effects present in the screen that are not well predicted by the baseline.

**Table 1:** Results from the cross-validation

			Latent GP		Dose response	
	$r_d$	$r_c$	wRMSE	Pearson’s *r*	wRMSE	Pearson’s *r*
PIICM	200	10	**0.3767**	**0.5336**	**0.0680** (0.0724)	**0.9800** (0.9733)
PIICM (no invariance)	100	10	1.1231	0.1895	0.1857 (**0.0724**)	0.8815 (**0.9733**)
PIICM (no measurement error)	200	10	1.6459	0.1731	0.2120 (**0.0724**)	0.8349 (**0.9733**)

Results are shown for prediction in the latent space, as well as for the resulting prediction of dose response. Corresponding results on dose–response for the baseline model are shown in parentheses for each setting. For both the latent GP and the dose–response, we compare our predictions against estimated values obtained from each experiment’s raw viability measurements using the bayesynergy R package. Bold numbers indicate best performance

**Fig. 4:**
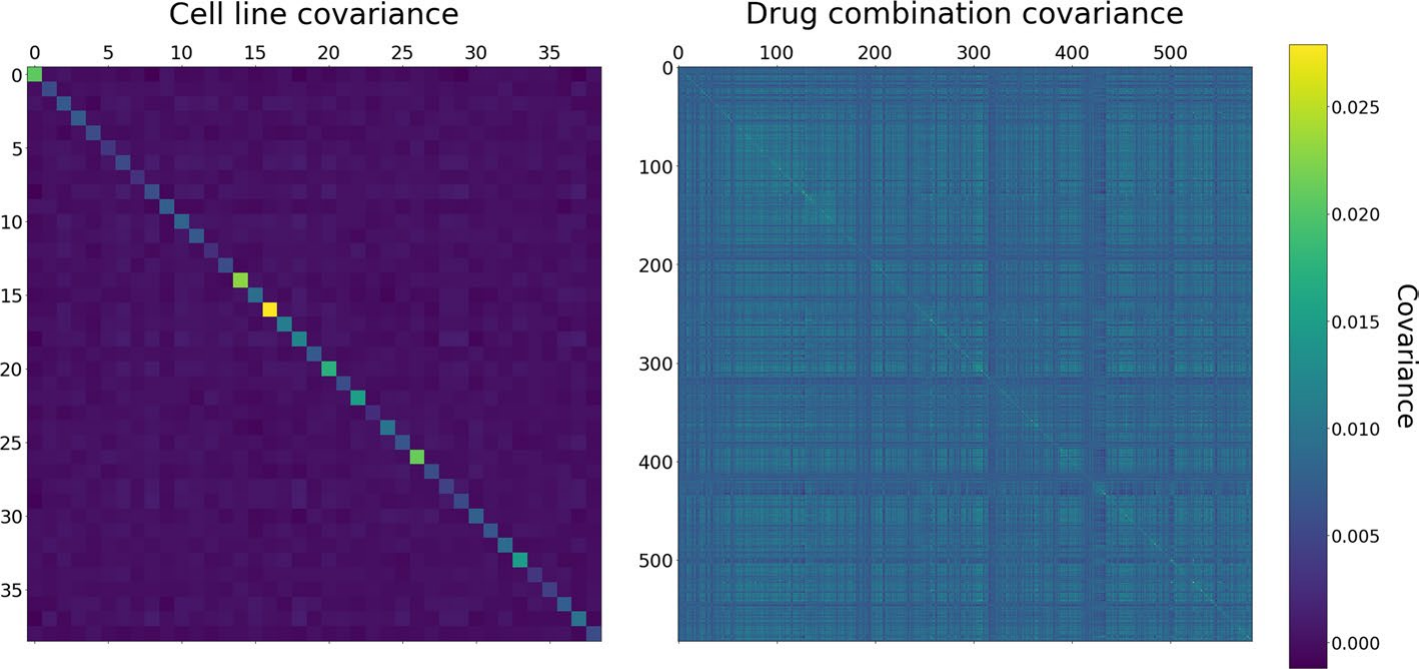
The figure shows the estimated cell line covariance matrix $K_c$ (left), and the overall drug combination covariance matrix $K_{d,\text{total}}$ (right), constructed by averaging $K_d$ across its blocks corresponding to the different possible orderings of the drug combinations

While the model is able to beat the baseline on held out experiments, it is not drastically better than simply using the Bliss non-interaction assumption. Part of the reason for this poor performance can be explained by looking at the estimated cell line and drug combination covariance matrices. In Fig. 4, the estimated matrices $K_c$ and $K_d$ are visualized. The estimated drug combination covariance matrix $K_d$ has a $2\times 2$ block structure $$K_d=\big ({\begin{matrix}A &{} B \\ B &{} C\end{matrix}}\big )$$, where *A* corresponds to the covariance between all the alphabetical orderings of drug pairs, *C* the covariance between the alphabetically reversed pairs, and finally *B* containing the cross-covariance. For the purposes of the figure, we display the $N_d \times N_d$ matrix $K_{d,\text{total}}=0.25(A+C+2B)$, obtained by averaging over the blocks – summarizing the drug combination covariance across both orderings of the drugs in a pair. This means that the plot visualizes the average covariance between drug combinations (*A*, *B*) and $(A',B')$ by looking at all possible orderings of the two pairs, i.e.

$$\begin{aligned}{} & {} ((A,B),(A',B')),((A,B),(B',A')),\\{} & {} ((B,A),(A',B')),((B,A),(B',A')). \end{aligned}$$

For the cell line covariance, most entries of the final matrix are near zero, with the exception of a strong diagonal containing cell line variances. This indicates minimal sharing of information across cell lines, and the model is essentially treating each cell line as an independent entity. For the drug combination covariance the situation is different, the matrix displaying many non-zero entries off the diagonal, and information is shared across combinations.

### Accounting for invariance and measurement error boosts performance

The model developed in this paper is specifically tailored for dose–response prediction in two major ways. First, we make use of the potentially large differences in estimation uncertainty, both within and between experiments, to give observations different weights in the final model through the estimation errors ${\textbf{S}}$. Secondly, we directly encode the relevant invariances for dose–response functions into the model through the kernel construction. This enables the model to learn from the data no matter which order the drug combinations are introduced in the training dataset. In this section we show that abandoning any of these two features has a negative impact on model performance.

We consider two distinct variations of the model. First, we assess the performance of a variation of the model that does not take into account the natural invariances of dose–response functions. The non-invariant version of the multi-output GP is simply the function ${\tilde{\zeta }}(\cdot )$ from Eq. (12) used to construct the final invariant $\zeta (\cdot )$. For a fully observed dataset, the kernel matrix takes the shape of a simple Kronecker product:

35 $$\begin{aligned} K = K_c\otimes K_d \otimes K_x. \end{aligned}$$

We utilize the same technique for inference and parameter learning as described in the “Inference and parameter learning” section using the optimal ranks for the drug combination and cell line covariance matrices found by CV in the previous section. In order to compensate for the lower dimensions of the drug combination space, we halve the rank of the drug combination kernel from the optimal 200 selected in the previous section to 100. The results are shown in Table 1, where in terms of the latent GP, prediction performance deteriorates from 0.3767 to 1.1231 in wRMSE and from 0.5336 to 0.1895 in correlation. The performance drop in the latent GP carries over to the dose–response, where wRMSE deteriorates from 0.0680 to 0.1857 and correlation from 0.98 to 0.8815, considerably worse than the baseline.

In the second variation, we remove the implicit weighting of observations through individual observation noise, but keep the model invariance. That is, we follow the regular inference procedure but set ${\textbf{S}}={\textbf{0}}$ for the observed entries, essentially giving all observations the same weight. Again, the corresponding results for this variation are shown in Table 1. Perhaps unsurprisingly, in terms of wRMSE the performance is worse than both the regular setting 1, and the non-invariant variation (1.6459 for latent GP, 0.2120 for dose–response). By treating all observations as equally precisely measured, the model is not allowed to ignore potential outliers or spurious large effects. The consequence of this is that the model attempts to fit all observations equally well, which in terms of the wRMSE means that contributions with low weight are given the same importance as contributions with high weight. This can have a detrimental effect on the model’s ability to accurately learn the required drug combination and cell line covariances. One might think that the correlation score would improve, as it does not depend on a weighting of the observations, but also here we see a decrease in model performance (Pearson’s r decreases from 0.5336 to 0.1731 in the latent GP, and from 0.9800 to 0.8349 in dose–response – worse than the baseline).

### Accurate reconstruction of synergistic dose–response surfaces

The plots in Fig. 3, and the metrics reported in Table 1 give an overall measure of the predictive performance of the model, across all drug concentrations and all outputs in the test set. In this section, we focus on a single experiment, and demonstrate that the model is able to accurately reconstruct the dose–response surface in a held-out experiment, and can capture relevant features indicating synergistic drug interaction.

Specifically, we inspect the prediction of the dose–response function in the experiment consisting of the two drugs Sorafenib and Vorinostat (Zolinza) on the lung cancer cell line MSTO. Sorafenib is a multi-kinase inhibitor (including RAF kinases and several receptor tyrosine kinases), while Vorinostat is an HDAC inhibitor. These two drugs are known in the literature to be highly synergistic in-vitro [51, 52], and have also been tested in a phase-I clinical trial for the treatment of patients with advanced hepatocellular carcinoma [53].

**Fig. 5:**
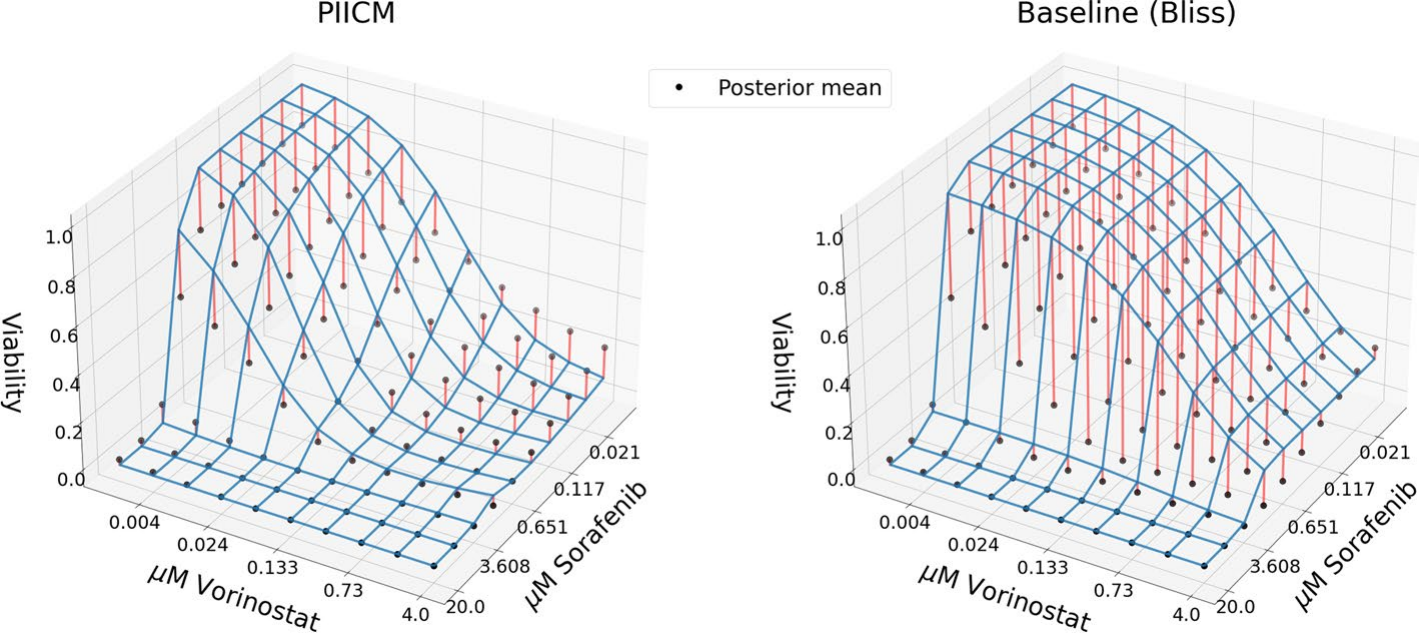
The predicted dose–response function for the two drugs Vorinostat and Sorafenib on the MSTO lung cancer cell line is displayed both the for PIICM model (left) and the baseline model (right). The red lines indicate the distances between the estimated dose–response values and the predicted surface. The estimated dose–response values (black dots) correspond to the posterior mean of the dose–response function, estimated using the bayesynergy R package, and collected in the matrix ${\textbf{F}}.$

The predicted dose–response function is shown in Fig. 5, where the left panel displays the estimated dose–response function using our model, and the right panel shows the corresponding prediction made by the baseline, i.e. only utilizing the Bliss non-interaction assumption. The red lines in the plot indicate the distances between the predicted dose–response, and the corresponding entries of ${\textbf{F}}$. We see that our model is able to fit the data much better than the baseline, and can adapt to the large synergistic effect of the two drugs. This is also reflected in the wRMSE computed only for this experiment, which is 0.1187 for the PIICM and 0.3337 for the baseline model.

### Predicting raw viability and comparing against comboLTR

We compare our modelling framework against comboLTR in terms of how well it can predict raw viability measurements. Unlike the previous sections, where the focus was to predict the dose–response function, *f*, of an unseen experiment, here we attempt to predict the actual viability measurements. We compare our model against the comboLTR model, and use the RMSE as the main metric of performance. The results are displayed in Table 2, where we compare the PIICM against the baseline (Bliss) model, as well as comboLTR. We see that in terms of RMSE, both comboLTR (RMSE = 0.1271) and PIICM (RMSE = 0.1270) outperform the Baseline model (RMSE = 0.1402), and that PIICM is marginally better at predicting viability compared to comboLTR. When looking at Pearson correlation, the results are similar, where PIICM is slightly outperforming comboLTR, with a Pearson’s r of 0.9267 against 0.9260.

**Fig. 6:**
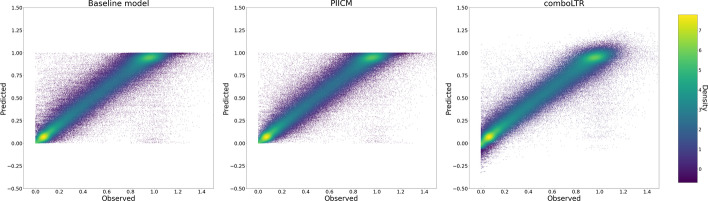
The figure shows observed versus predicted viability measurements for the baseline (Bliss) model (left panel), the PIICM (middle panel), and the comboLTR model (right panel). The points have been coloured according to the density of points. Overall, the performance of the three models are similar, with the main differences being how the models handle observations outside the [0, 1] interval. Both the baseline and PIICM are limited to prediction within this interval, while the comboLTR is not

The results are also shown in Fig. 6, where we plot the observed viability measurements against the predicted viabilities from the baseline, PIICM and comboLTR models. The most noticable feature of the figure is the hard limits on the prediction by the baseline model and the PIICM. These models never predict viability measurements outside the [0, 1] interval, assuming these viability measurements to represent biological and technical noise. This reflects the underlying assumption that no cancer drugs can be beneficial to cell growth, nor can the drugs induce cell death below zero per cent viability. The comboLTR model does not have such a restriction, and will predict viability measurements outside this bound. Part of the reason for the similar performance of comboLTR and PIICM in terms of RMSE and correlation despite the plots looking dissimilar, is that most viability measurements are within the [0, 1] range, in particular measurements below zero are very rare. Also note the persistence of viability measurements near one, with varying prediction accuracy in the lower right corner, as was observed also in Fig. 3. That these observations are still not predicted well could indicate that they are outliers that should be removed in pre-processing.

**Table 2:** Results showing the prediction performance of the baseline (Bliss) model, PIICM, and comboLTR in predicting raw viability measurements

	Viability	
	RMSE	Pearson’s *r*
Baseline (Bliss)	0.1402	0.9103
PIICM	**0.1270**	**0.9267**
comboLTR	0.1271	0.9260

Bold numbers indicate best performance

## Conclusion

In this paper we developed a novel approach to predicting dose–response for in-vitro drug combination experiments on cancer cell lines. We constructed a permutation invariant multi-output Gaussian process prior that encodes the symmetries and invariances inherent in dose–response functions. A two-stage observation model was utilized that introduces individual level observation noise, accounting for the inherent measurement errors in the dose–response experiments. These measurement errors reflect both the natural uncertainty across the dose–response surface *within* an experiment (due to e.g. the stochasticity of cell growth, or technical errors from the viability assay), but additionally they capture differences in uncertainty *between* experiments, which can be substantial depending on e.g. the number of technical replicates or the design of the experiment. Utilizing the conjugacy of the model and Kronecker algebra, we described a fast, scalable and exact inference procedure that handles incomplete datasets which are commonplace in drug combination screens. We showed that our model outperforms the baseline model (Bliss independence) when predicting the dose–response function of untested experiments, and that accounting for both measurement error and invariance is crucial for the model’s performance. Furthermore, we illustrated that the model can predict synergistic dose–response surfaces in held-out experiments.

The model in this paper can be extended in numerous ways, the most readily available being different kernel choices for the drug combination and cell line covariances. While the low-rank structure currently utilized offers a lot in terms of flexibility to learn complex patterns of covariance, this choice also have some downsides.

Firstly, the model cannot predict dose–response in new drug combinations or cell lines that are not already part of the training dataset. Prediction in a GP setting requires the computation of the covariance between the test and training data. Thus, for a new cell line $c_*$, we need to compute the covariance between the new cell line and the cell lines already observed in the training data, $\kappa _c(c_*,c_i)$ for $i=1,\ldots ,N_c$. Since the low-rank kernels are completely non-parametric, there are no covariates linking the new cell line to the cell lines in the training data. Thus, the required terms cannot be computed and the model cannot predict dose–response in new cell lines or for new drug combinations. Secondly, the ranks of the kernel matrices must be determined using cross-validation, which is computationally expensive. Thirdly, the low-rank structure needs a lot of parameters. For example, for $N_d=583$ and $r_d=200$, the kernel matrix $K_d$ depends on over 200 thousand parameters. This has the effect of slowing down parameter learning through the marginal likelihood, as well as the model struggling to converge to sensible locations in the parameter space. This could explain why the estimated cell line covariance in Fig. 4 is essentially a diagonal, and is close to its initialization value.

Since the model is built within the GPyTorch framework, it is highly modular and easily customizable. Swapping out the low-rank kernels for a different construction would be straight forward, requiring minimal changes to the underlying code or inference procedures. This would allow the model to include relevant auxiliary data regarding the drug combinations and cancer cell lines, which in turn would make it possible to predict dose–response for previously unseen cell lines and combinations. One avenue to explore in this direction is to utilize multiple kernel learning (MKL) [54] to construct the drug combination and cell line covariances, which has previously been utilized successfully for drug response prediction in the single-drug case [37].

Adding external data regarding the cell lines, such as various *omics* characterizations, or tissue indicators, is likely to increase the prediction performance of the model, as previously observed by numerous authors [12, 14, 22]. In particular, it might help guide the model towards more sensible areas of the parameter space and encourage the model to borrow information across the cell lines, instead of treating each cell line as independent of each other. Through the kernels, various chemical descriptors can be utilized as well. For example, chemical structure information regarding the drugs [55], or information regarding their targets [56]. Overall, this will have the impact of greatly reducing the number of parameters in the model, adding further regularization to a learning problem where the data is scarce and noisy, helping the model’s generalizability to unseen experiments.

## Supplementary Information

**Additional file 1**. Supplementary material. Contains a derivation of the invariant kernel, a proof of Proposition 1, full model specification of the bayesynergy model used for pre-processing, and a comparison with the IDACombo framework.

## Data Availability

The software implementation of the model named PIICM is available at project home page https://github.com/ltronneb/PIICM, archived version v1.0, DOI:10.5281/zenodo.6366091. The software is platform-independent and uses programming languages R and Python (requirements: Python $>=$ 3.6, PyTorch $>=$ 1.8.1). The software is licensed under MIT license. There are no further restrictions for use by non-academics except those specified by the license.
