# Supplementary material for: Dose–response prediction for in-vitro drug combination datasets: a probabilistic approach
Source: BMC Bioinformatics. 2023 Apr 21;24:161. doi: 10.1186/s12859-023-05256-6 (PMC10120211; doi:10.1186/s12859-023-05256-6)
Supplement: Supplementary file 1 — Additional file 1. Supplementary material. Contains a derivation of the invariant kernel, a proof of Proposition 1, full model specification of the bayesynergy model used for pre-processing, and a comparison with the IDACombo framework. [file 12859_2023_5256_MOESM1_ESM.pdf]

Dose-response prediction for *in-vitro* drug combination datasets: a probabilistic approach

Supplementary Material

Leiv Rønneberg, Paul D. W. Kirk, Manuela Zucknick

S1 Derivation of the invariant kernel

By using that

$$\begin{aligned} z(c,A,B,x_1,x_2) &= \tilde{z}(c,A,B,x_1,x_2) + \tilde{z}(c,B,A,x_2,x_1), \\ \tilde{z}(c,A,B,x_1,x_2) &\sim \mathcal{GP}\left(0, \kappa_c(c,c')\kappa_d((A,B),(A',B'))\kappa_x((x_1,x_2),(x_1',x_2'))\right), \end{aligned} \tag{1}$$

and that $\mathrm{Cov}\left[X,Y\right] = \mathbb{E}\left[XY\right] - \mathbb{E}\left[X\right]\mathbb{E}\left[Y\right]$ we have

$$\mathrm{Cov}\left[z(c,A,B,x_1,x_2), z(c',A',B',x_1',x_2')\right] = \mathbb{E}\left[z(c,A,B,x_1,x_2)z(c',A',B',x_1',x_2')\right],$$

since the GP has mean zero. Hence, by substituting in the sum formulation of $z(c,A,B,x_1,x_2)$, and defining the shorthands $\mathbf{x} := (x_1,x_2)$ and $\tilde{\mathbf{x}} := (x_2,x_1)$ we obtain

$$\begin{aligned} &\mathbb{E}\left[\left(\tilde{z}(c,A,B,\mathbf{x}) + \tilde{z}(c,B,A,\tilde{\mathbf{x}})\right)\left(\tilde{z}(c',A',B',\mathbf{x}') + \tilde{z}(c',B',A',\tilde{\mathbf{x}}')\right)\right] \\ =&\mathbb{E}\left[\tilde{z}(c,A,B,\mathbf{x})\tilde{z}(c',A',B',\mathbf{x}')\right] + \mathbb{E}\left[\tilde{z}(c,A,B,\mathbf{x})\tilde{z}(c',B',A',\tilde{\mathbf{x}}')\right] \\ +&\mathbb{E}\left[\tilde{z}(c,B,A,\tilde{\mathbf{x}})\tilde{z}(c',A',B',\mathbf{x}')\right] + \mathbb{E}\left[\tilde{z}(c,B,A,\tilde{\mathbf{x}})\tilde{z}(c',B',A',\tilde{\mathbf{x}}')\right] \\ =&\mathrm{Cov}\left[\tilde{z}(c,A,B,\mathbf{x}), \tilde{z}(c',A',B',\mathbf{x}')\right] + \mathrm{Cov}\left[\tilde{z}(c,A,B,\mathbf{x}), \tilde{z}(c',B',A',\tilde{\mathbf{x}}')\right] \\ +&\mathrm{Cov}\left[\tilde{z}(c,B,A,\tilde{\mathbf{x}}), \tilde{z}(c',A',B',\mathbf{x}')\right] + \mathrm{Cov}\left[\tilde{z}(c,B,A,\tilde{\mathbf{x}}), \tilde{z}(c',B',A',\tilde{\mathbf{x}}')\right] \\ =&\kappa_c(c,c')\left[\kappa_d((A,B),(A',B'))\kappa_x(\mathbf{x},\mathbf{x}') + \kappa_d((B,A),(B',A'))\kappa_x(\tilde{\mathbf{x}},\tilde{\mathbf{x}}') + \right. \\ &\left. \kappa_d((A,B),(B',A'))\kappa_x(\mathbf{x},\tilde{\mathbf{x}}') + \kappa_d((B,A),(A',B'))\kappa_x(\tilde{\mathbf{x}},\mathbf{x}')\right]. \end{aligned}$$

Furthermore, $k_x(\mathbf{x},\mathbf{x}') = k_x(\tilde{\mathbf{x}},\tilde{\mathbf{x}}')$ and $k_x(\mathbf{x},\tilde{\mathbf{x}}') = k_x(\tilde{\mathbf{x}},\mathbf{x}')$ due to the symmetric properties of the kernel function and since reflection is a linear operator. Thus we can further simplify the kernel to

$$\begin{aligned} \kappa_c(c,c')\left[\left(\kappa_d((A,B),(A',B')) + \kappa_d((B,A),(B',A'))\right)\kappa_x(\mathbf{x},\mathbf{x}') + \right. \\ \left.\left(\kappa_d((A,B),(B',A')) + \kappa_d((B,A),(A',B'))\right)\kappa_x(\tilde{\mathbf{x}},\mathbf{x}')\right]. \end{aligned}$$

If this kernel was further simplified, e.g. by insisting also that the covariance of the drug pairs could be reflected such that the covariance of the pairs (A,B) and (B,A) were identical, the kernel would take the form:

$$2\kappa_c(c,c')\kappa_d((A,B),(A',B'))\left[\kappa_x(\mathbf{x},\mathbf{x}') + \kappa_x(\tilde{\mathbf{x}},\mathbf{x}')\right], \tag{2}$$

and would enforce the restriction

$$z(c,A,B,\mathbf{x}) = z(c,A,B,\tilde{\mathbf{x}}).$$

That is, all dose-response surfaces would be forced to be symmmetric around the 45°line. When constructing the drug combination kernel, care must be taken to ensure that the two pairs (A,B) and (B,A) are considered different entities, otherwise the kernel collapses to (2)

S2 Proof of Proposition 1

In this section we prove Proposition 1 from the main paper. The proof hinges on some properties of matrices that can be written in a specific way, namely as

$$S = (A + QAQ) + (QA + AQ), \tag{3}$$

where Q is a symmetric permutation matrix and A a positive semi-definite (PSD) matrix. The following lemma will be of use in the proof

Lemma 1. *Define $M_1 = (A + QAQ)$, $M_2 = (QA + AQ)$ and $S = M_1 + M_2$, where Q is a symmetric permutation matrix and A a positive semi-definite matrix. Then the following properties hold*

i. *$M_1 = QM_2 = M_2Q$, $M_2 = QM_1 = M_1Q$ and $QS = SQ$.*

ii. *S, M_1 and M_2 have the same eigenvectors. Moreover, if v is an eigenvector with eigenvalue λ_1 for M_1 and λ_2 for M_2, it will have eigenvalue $\lambda_1 + \lambda_2$ for S.*

iii. *If v is an eigenvector with non-zero eigenvalues λ_1 for M_1 and λ_2 for M_2 then*

$$\lambda_1 = \lambda_2 \Longleftrightarrow v = Qv.$$

iv. *If v is an eigenvector with eigenvalue λ for S, and eigenvalues λ_1 for M_1 and λ_2 for M_2, then either:*

- *$\lambda = 0$, in which case $\lambda_1 = -\lambda_2$; or*
- *$\lambda \neq 0$, in which case $\lambda_1 = \lambda_2$.*

v. *S is PSD. Furthermore, let $\sigma(S)^+$ denote the set of all positive eigenvalues of S, then*

$$\sigma(S)^+ = \sigma(2M_2)^+. \tag{4}$$

Proof.

i) Trivial using properties of symmetric permutation matrices, $Q = Q^{-1} = Q^T$.

ii) Since both M_1 and M_2 are symmetric, they are diagonalizable, i.e. $M_1 = V_1 D_1 V_1^T$ and $M_2 = V_2 D_2 V_2^T$, where V_i is the matrix whose columns are eigenvectors for M_i and D_i is a diagonal matrix containing the corresponding eigenvalues. Furthermore, it is easy to check that the matrices M_1 and M_2 commute, i.e. $M_1M_2 = M_2M_1$ and hence they are *simultaneously diagonalizable* [1, Theorem 1.3.12]:

$$S = V(D_1 + D_2)V^T,$$

where $V = V_1 = V_2$. The result now follows.

iii) "$\Rightarrow$": Suppose $\lambda_1 = \lambda_2 = \lambda$ and that $\lambda \neq 0$, then $\lambda v = M_1 v = M_2 v = QM_1 v = \lambda Qv$, since $M_2 = QM_1$. Dividing through by λ gives $v = Qv$.
"$\Leftarrow$": Suppose $v = Qv$. Then $\lambda_1 v = M_1 v = M_2 Qv = M_2 v = \lambda_2 v$, and hence $\lambda_1 = \lambda_2$, noting that $v \neq 0$ since it is an eigenvector.

iv) Suppose $\lambda = 0$. Then by (ii) we have $\lambda_1 + \lambda_2 = 0$, and hence $\lambda_1 = -\lambda_2$ directly.

Suppose $\lambda \neq 0$. Since v is the corresponding eigenvector, $Sv = \lambda v$ and hence $QSv = \lambda Qv$. Using that $QS = SQ$ we obtain $SQv = \lambda Qv$, hence either $Qv = 0$ (ruled out since v is an eigenvector) or Qv is an eigenvector of S with eigenvalue λ. Now, since both $SQv = \lambda Qv$ and $Sv = \lambda v$, we can write $S(Qv - v) = \lambda(Qv - v)$. Thus $(M_1 + M_2)(Qv - v) = \lambda(Qv - v)$, and

$$\begin{aligned}\lambda(Qv - v) &= (M_1 + M_2)(Qv - v) \\ &= M_1Qv - M_1v + M_2Qv - M_2v \\ &= M_2v - M_1v + M_1v - M_2v \\ &= 0.\end{aligned}$$

Since $\lambda \neq 0$ by assumption, this implies that $Qv - v = 0$. Now since M_1 and M_2 have the same null space (since $M_1 = QM_2$), both λ_1 and λ_2 have to be non-zero, and hence by (iii) $\lambda_1 = \lambda_2$.

v) Since S and M_2 have common eigenvectors according to (ii), it suffices to show for any eigenvector v how each corresponding eigenvalue of M_2 is related to the corresponding eigenvalue of S. Let v denote an eigenvector, with eigenvalue λ for S, λ_1 for M_1 and λ_2 for M_2. There are three cases to consider.

   First, suppose that $\lambda_2 = 0$ Then, since M_2 and M_1 have the same null space (since $M_1 = QM_2$), $\lambda_1 = 0$ and from (ii) also $\lambda = 0$.

   Next, suppose $\lambda_2 > 0$. Then from (ii) and using that M_1 is PSD, $\lambda > 0$. Now from (iv) $\lambda_1 = \lambda_2$ and thus from (ii) $\lambda = 2\lambda_2$

   Finally, suppose $\lambda_2 < 0$. If we now assume that $\lambda \neq 0$, then from (iv) we have $\lambda_2 = \lambda_1 < 0$, contrary to M_1 being PSD. Thus $\lambda = 0$.

   Thus for any eigenvector v of S, the corresponding eigenvalue is either zero or $2\lambda_2$ if λ_2 is positive, this S is PSD and the result follows.

□

Proposition 1. *Let K_c, K_d and K_x denote positive semi-definite matrices, while P and $\tilde{P}$ denote symmetric permutation matrices. Furthermore, let $\sigma(A)^+$ denote the collection of positive eigenvalues of the matrix A. Then, the matrix*

$$K = K_c \otimes \left[(K_d + PK_dP) \otimes K_x + (PK_d + K_dP) \otimes \tilde{P}K_x \right],$$

is positive semi-definite (PSD) and

$$\sigma(K)^+ = 2\sigma(K_c \otimes (PK_d + K_dP) \otimes \tilde{P}K_x)^+$$

Proof. First note that since the eigenvalues of a Kronecker product $A \otimes B$ is simply the product of all eigenvalues of A by all eigenvalues of B, it suffices to prove that the eigenvalues of

$$S = (K_d + PK_dP) \otimes K_x + (PK_d + K_dP) \otimes \tilde{P}K_x \tag{5}$$

are exactly the positive eigenvalues of $(PK_d + K_dP) \otimes \tilde{P}K_x$. Now, the proposition can be proved by simply showing that S can be written such that Lemma 1 applies.

By using the mixed-product property of Kronecker products, $(A \otimes B)(C \otimes D) = (AC) \otimes (BD)$, and the identities $K_x = \tilde{P}K_x\tilde{P}$ and $\tilde{P}K_x = K_x\tilde{P}$ (since $\kappa_x(\tilde{\mathbf{x}}, \mathbf{x}')$ is symmetric) we can rewrite

$$S = (K_d + PK_dP) \otimes K_x + (PK_d + K_dP) \otimes \tilde{P}K_x = (C + QCQ) + (QC + CQ), \tag{6}$$

where $Q = (P \otimes \tilde{P})$ is a symmetric permutation matrix and $C = (K_d \otimes K_x)$ is a positive semi-definite matrix. Hence the result follows from Lemma 1(v). □

S3 Full model description for single experiment dose-response

In this section, we provide the full model description used to fit each individual experiment to the dose-response model in section **??**. The model is provided solely for reference, and we refer to the original paper [2] for an extensive discussion of the model.

The bayesynergy model takes as takes as input cell viability measurements $\mathbf{y} = (y_1, \ldots, y_n)$, measured at a corresponding set of drug concentrations $\mathbf{X} = (\mathbf{x}_1, \ldots, \mathbf{x}_n)$. Typically, drug combination experiments are performed on a grid of concentrations, and we could assume that $\mathbf{X} = X_1 \times X_2$, where $X_1 = (x_1, \ldots, x_{n_1})$ denote the n_1 concentrations of drug 1 and $X_2 = (x_2, \ldots, x_{n_2})$ the n_2 concentrations of drug 2. The experiment may also include technical replicates, where cell viability have been measured multiple times at each drug concentration. We can assume that the dataset contains n_{rep} technical replicates, and thus the measured viabilities can be denoted by y_{ijr} where $i = 1, \ldots, n_1$, $j = 1, \ldots, n_2$ and $r = 1, \ldots, n_{rep}$. A heteroskedastic noise model is assumed, that depends on the value of the dose-response function f at the

corresponding concentration, and the GP prior is given a Matérn-3/2 covariance function. The complete model description is as follows

$$
\begin{aligned}
y_{ijr} &\sim \mathcal{N}\left(f(\boldsymbol{x}_{ij}), \sigma^2(f(\boldsymbol{x}_{ij})+\lambda)\right), \quad \mathbf{x}_{ij}=(x_{1i},x_{2j}) \\
i &= 1,\dots,n_1,\ j=1,\dots,n_2,\ r=1,\dots,n_{\text{rep}}, \\
\sigma &\sim \text{Inv-Gamma}\,(5,1)\,,\ \lambda = 0.005. \\
f(\boldsymbol{x}_{ij}) &= p_0(\boldsymbol{x}_{ij}) + \Delta(\boldsymbol{x}_{ij})\mathbb{I}(10^{\boldsymbol{x}_{ij}}>0) \\
p_0(\boldsymbol{x}_{ij}) &= h_1(x_{1i}|l_1,s_1,m_1)\ h_2(x_{2j}|l_2,s_2,m_2). \\
h_k(x_{ki}) &= l_k + \frac{1-l_k}{1+10^{s_k(x_{ki}-m_k,)}}\ \ k=1,2 \\
l_k &\sim \text{Beta}(1,1.25),\ s_k \sim \text{Gamma}(1,1) \\
m_k &\sim \mathcal{N}(\theta_k,\sigma^2_{m_k}),\ k=1,2 \\
\theta_k &\sim \mathcal{N}(0,1),\ \sigma^2_{m_k} \sim \text{Inv-Gamma}\,(3,2)\,,\ k=1,2 \\
\Delta(\boldsymbol{x}_{ij}) &= g(z(\boldsymbol{x}_{ij})),\ z(\boldsymbol{x}_{ij}) \sim \mathcal{GP}(0,\kappa(\boldsymbol{x}_{ij},\boldsymbol{x}_{i'j'})) \\
g(z(\boldsymbol{x}_{ij})) &= \frac{-p_0(\boldsymbol{x}_{ij})}{1+\exp\left\{b_1 z(\boldsymbol{x}_{ij}) + \log\left[\frac{p_0(\boldsymbol{x}_{ij})}{1-p_0(\boldsymbol{x}_{ij})}\right]\right\}} \\
&+ \frac{1-p_0(\boldsymbol{x}_{ij})}{1+\exp\left\{-b_2 z(\boldsymbol{x}_{ij}) - \log\left[\frac{p_0(\boldsymbol{x}_{ij})}{1-p_0(\boldsymbol{x}_{ij})}\right]\right\}} \\
\kappa(\boldsymbol{x}_{ij},\boldsymbol{x}_{i'j'}) &= \sigma_f^2\left(1+\frac{\sqrt{3}\|\boldsymbol{x}_{ij}-\boldsymbol{x}_{i'j'}\|}{\ell}\right)\exp\left\{-\frac{\sqrt{3}\|\boldsymbol{x}_{ij}-\boldsymbol{x}_{i'j'}\|}{\ell}\right\}, \\
\sigma_f^2 &\sim \text{log-}\mathcal{N}(1,1),\ \ell \sim \text{Inv-Gamma}(5,5) \\
b_1 &\sim \mathcal{N}(1,0.1^2),\ b_2 \sim \mathcal{N}(1,0.1^2).
\end{aligned}
\tag{7}
$$

Note that the implementation of this model in the bayesynergy R package does not need a complete grid of observations, nor a complete set of replications. It takes as input a set of cell viability measurements $\mathbf{y}$ and the corresponding drug concentrations $\mathbf{X}$ and provides samples from the posterior distribution of the model at each drug concentration $\mathbf{x}$ in $\mathbf{X}$.

We use this approach to fit each dose-response experiment, and draw samples from the posterior distribution of the latent GP $z(\mathbf{x})$, and the dose-response function $f(\mathbf{x})$, at drug concentration $\mathbf{x}$. If samples are needed at a location $\mathbf{x}_*$ not observed in the original dataset, $z(\mathbf{x}_*)$ and $f(\mathbf{x}_*)$ are sampled from the posterior predictive distribution. From these samples, we compute posterior means and variances, which are used to construct the matrices $\mathbf{Z}$, $\mathbf{S}$ for the latent GP, and $\mathbf{F}$ and $\mathbf{S}_F$ for the dose-response function.

Additionally, we use a simplified version of this model to estimate the individual monotherapies for each drug on every cell line. These are used to construct the estimate $\hat{p}_{0*}$ in equation (**??**) of the main text. Given a monotherapy dataset of cell viabilities $\mathbf{y}=(y_1,\dots,y_n)$ measured at concentrations $\mathbf{X}=(x_1,\dots,x_n)$, we estimate the following model

$$
\begin{aligned}
y_{ir} &\sim \mathcal{N}\left(h(x_i), \sigma^2(h(x_i)+\lambda)\right), \\
i &= 1,\dots,n,\ r=1,\dots,n_{\text{rep}}, \\
\sigma &\sim \text{Inv-Gamma}\,(5,1)\,,\ \lambda = 0.005. \\
h(x_i) &= l + \frac{1-l}{1+10^{s(x_i-m,)}} \\
l &\sim \text{Beta}(1,1.25),\ s \sim \text{Gamma}(1,1) \\
m &\sim \mathcal{N}(\theta_k,\sigma^2_{m_k}), \\
\theta &\sim \mathcal{N}(0,1),\ \sigma^2_m \sim \text{Inv-Gamma}\,(3,2)\,,
\end{aligned}
\tag{8}
$$

for each drug combination on every cell line. For any two drugs on any cell line, we can then compute a sample from the predictive posterior distribution of $p_0(\mathbf{x}_*)$ by first sampling the individual monotherapy

evaluations $h_1(x_{1*})$ and $h_2(x_{2*})$ from their individual posterior predictive distributions and constructing $p_0(\mathbf{x}_*) = h_1(x_{1*})h_2(x_{2*})$. The estimate $\hat{p}_{0*}$ can then be computed by averaging across all samples.

S4 Comparison with IDACombo

We include here a short comparison of the PIICM modelling framework with IDACombo [3]. The IDACombo framework is based on the notion of Independent Drug Action (IDA), which hypothesises that most drugs act independently of each other and do not interact. In fact, the IDA principle states that the expected effect of a drug combination is exactly equal to the effect of the single most effective drug of the combination administered on its own. This principle is sometimes referred to as the *highest single agent* (HSA) in synergy modelling. Predictions with IDACombo for drug combination (A, B) at concentration $\mathbf{x} = (x_A, x_B)$ is made by first finding the minimum of the two monotherapy functions for the drugs, i.e. $\min\{h_A(x_A), h_B(x_B)\}$. Then this is done for each of the $i = 1, \ldots, n_c$ cell lines in the training dataset and these are averaged to produce a final prediction of expected dose-response, i.e.:

$$\mu_{(A,B,\mathbf{x}),\mathrm{IDA}} = \frac{\sum_{i=1}^{n_c} \min\{h_{A,i}(x_A), h_{B,i}(x_B)\}}{n_c},$$

where the monotherapy functions $h_{A,i}$ and $h_{B,i}$ denote the monotherapy functions of drugs A and B on the ith cell line, respectively.

In many ways, this is similar to an underlying assumption of the PIICM, where we model dose-response as a two-component function, $f(\mathbf{x}) = p_0(\mathbf{x}) + \Delta(\mathbf{x})$, where f denotes the dose-response function taking as arguments the drug concentrations $\mathbf{x}$. The non-interaction component, p_0, captures our prior expectation regarding the behaviour of f, and while we prefer the Bliss non-interaction assumption for this – we could have just as easily chosen something else, like IDA. The interaction component, Δ, is modelled as a zero-mean GP pushed through a transformation function g that has the property that $g(0) = 0$. In terms of prior expectation, we have that $\mathbb{E}[f] \approx p_0$, for all $\mathbf{x}$. The PIICM is constructed to model everything in p_0 as a function of the monotherapy measurements, and focuses its attention on modelling whatever is left over by the multi-output GP. If the model becomes uncertain, or the GP is extrapolating outside the data range, the behaviour of the GP is such that predictions made with PIICM will be shrunken towards p_0.

The key difference in the two frameworks is that PIICM is focused on predicting the *deviations from* a non-interaction assumption, while IDACombo produces predictions that are *equal to* a non-interaction assumption. Another key difference is that IDACombo only produces an averaged predicted response, across multiple cell lines. This means that for a given drug combination and drug concentration, IDACombo will predict the same response value for all cell lines in the test dataset, while PIICM (and both comboFM and comboLTR) produce cell-line specific predictions.

While we can compare predictions made by IDACombo to PIICM or comboLTR, we should note that such a comparison is not fair to IDACombo. PIICM and comboLTR are both trained on actual combination data, and not only monotherapy measurements. In fact, it would be more sensible to compare IDACombo against simply the baseline Bliss model $\hat{p}_0$ utilized in the main results section. We show the results of such a comparison in Figure S1, which is a version of Figure 6 with predictions made by IDACombo. Using a similar approach as in the original IDACombo paper, we only evaluate the performance of the models at the maximum concentration tested for each drug in the combination. When predicting raw viability measurements, IDACombo performs poorly compared to both PIICM and the Bliss baseline model, with Pearson's correlation between observed and predicted at only 0.60 for IDACombo, compared to 0.83 for the baseline and 0.88 for PIICM. The poorer performance compared to the baseline is mainly due to the aforementioned property that IDACombo does not produce cell-line specific predictions. Following further the IDACombo paper, which report the average performance across cell lines, we compute the average of our predictions and observed viability measurements across all the cell lines in the test set. The results are visualized in Figure S2, which again shows the poorer performance of IDACombo compared to both the baseline and the PIICM model. Pearson's correlation between average observed and average predicted is 0.48 for IDACombo, 0.92 for the baseline, and 0.97 for PIICM. Again, this is mostly due to IDACombo not supplying a cell-line specific prediction, but rather a single number representing an average dose response

across cell lines. Given that synergy is quite rare, this averaged prediction might be an okay approximation to the average dose-response when the number of cell lines become large (as most cell lines will tend to follow e.g. the IDA principle), however it does not perform well in a small sample train-test split setting as here.

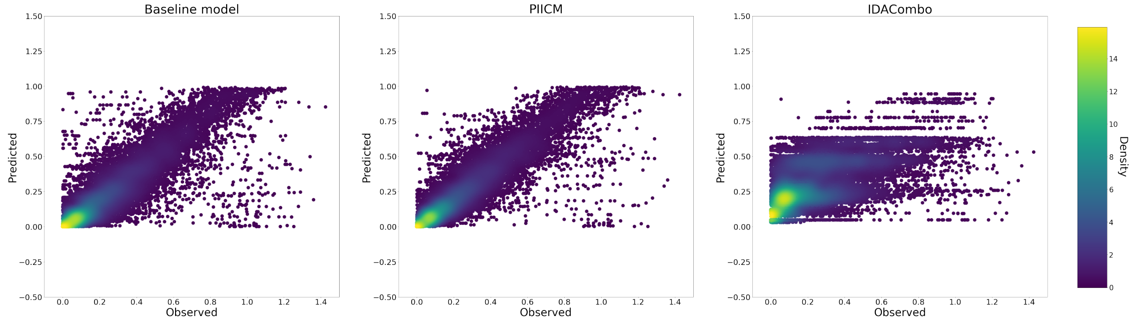

Figure S1: The figure shows predicted versus observed viability for the baseline, PIICM and the IDACombo models. Each dot in the figure represents a unique (cell line, drug A, drug B, conc. drug A, conc. drug B)-quintuplet, and is colored according to the density of points. Furthermore, the comparison has only been done using the maximum concentration for each drug in a combination. Overall, both the baseline model and PIICM have a comparatively large advantage over IDACombo in terms of predictive performance.

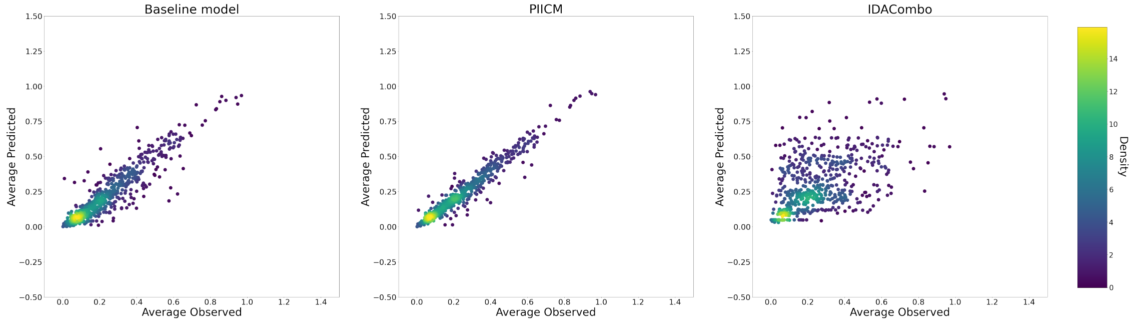

Figure S2: The figure shows the average predicted versus average observed viability for the baseline, PIICM and the IDACombo models. The average has been taken across the cell lines, and hence each dot in the figure represents a unique (drug A, drug B, conc. drug A, conc. drug B)-quadruplet, colored according to the density of points. We see the same pattern as before, with both the baseline model and PIICM outperforming IDACombo in terms of predictive performance.

References

[1] Roger A Horn and Charles R Johnson. *Matrix Analysis*. Cambridge University Press, Second edition, 2012.

[2] Leiv Rønneberg, Andrea Cremaschi, Robert Hanes, Jorrit M Enserink, and Manuela Zucknick. bayesynergy: flexible Bayesian modelling of synergistic interaction effects in in vitro drug combination experiments. *Briefings in Bioinformatics*, 22(6), 2021.

[3] Alexander Ling and R. Stephanie Huang. Computationally predicting clinical drug combination efficacy with cancer cell line screens and independent drug action. *Nature Communications*, 11(1), 2020.
